# Nuclear RNA clusters are dynamic structural entities in Huntington’s disease

**DOI:** 10.1038/s42003-026-10419-1

**Published:** 2026-06-29

**Authors:** Sandra Fienko, Iulia M. Nita, Ignacio Munoz-Munoz, Gillian P. Bates

**Affiliations:** Huntington’s Disease Centre and Department of Neurodegenerative Disease, Queen Square Institute of Neurology, University College London, London, UK

**Keywords:** Huntington's disease, Molecular neuroscience

## Abstract

Huntington’s disease research has focused on either loss of normal huntingtin function or toxic gain-of-function of the mutant huntingtin protein as a key driver of pathology. The role of mutant *HTT* mRNA in vivo has been only partially studied and remains largely unexplored. Recently, we discovered that full-length human *HTT* mRNA is retained, together with the alternatively processed *HTT1a* transcript, in RNA nuclear clusters in YAC128 mouse brains. Here, we demonstrate that these clusters were present at the prenatal stage, indicating early developmental effects. Moreover, these clusters were confined to neurons, implying a neuron-specific mechanism of accumulation, and were colocalised with core spliceosomal proteins, suggesting an impact on nuclear homeostasis. *HTT* nuclear RNA clusters showed remarkable dynamics, rapidly dissolving when ionic interactions were disrupted or transcription and splicing were inhibited. This malleability underscores the importance of *HTT* mRNA accessibility to therapeutic interventions and may guide future design of *HTT*-targeting therapies.

## Abstract (web summary)

Mutant human huntingtin mRNAs form dynamic nuclear clusters in neurons that associate with spliceosome components, highlighting a potential role for mutant HTT mRNAs in Huntington’s disease pathology and its accessibility to RNA-targeted therapeutics.

## Introduction

Huntington’s disease (HD) is a fatal neurodegenerative disorder, caused by an expanded CAG repeat in exon 1 of the huntingtin gene (*HTT*), that translates into a polyglutamine stretch in the huntingtin protein (HTT)^1^. Our previous data indicate that in the context of expanded CAG repeat, huntingtin pre-mRNA undergoes alternative processing to generate a small transcript comprising exon 1 and 5′ intron 1 sequences (*HTT1a*)^2^. This occurs when one of two cryptic polyadenylation (polyA) signals in intron 1 is activated. The extent of *HTT* pre-mRNA processing is determined by CAG repeat length, with a longer repeat producing greater *HTT1a* levels^3^. Importantly, *HTT1a* has not only been detected in numerous HD mouse models^2^, but it has also been observed in patient-derived fibroblast, human peripheral blood cells and post-mortem brains from HD mutation carriers^4,5^. *HTT1a* is directly relevant to disease pathogenesis and progression as it encodes the highly pathogenic and aggregation-prone exon 1 HTT protein (HTT1a). The toxicity of the HTT1a protein isoform is underscored by the R6/2 mouse model, which expresses this noxious fragment and represents the HD model with the most rapidly progressing phenotype^6^. Consistently, aggregates in human *post-mortem* tissue are stained with antibodies recognising N-terminal fragments of HTT^7^.

Recently, we discovered that fully processed human *HTT* mRNA is retained, together with the* HTT1a* transcript, in nuclear RNA clusters in the brains of 2-month-old YAC128 mice^8^. A subsequent study showed that this also occurs in BACHD-97Q-∆N17 mice as early as one month of age^9^. Together, these findings indicate that nuclear retention and clustering are features of human *HTT* transcripts that emerge early across HD models, well before overt pathology. Here, we extend these observations by demonstrating that *HTT* nuclear clusters are present even during prenatal brain development in YAC128 mice.

Huntingtin protein aggregation and its downstream detrimental effects on vital cellular processes have been extensively investigated. However, a growing literature suggests a role for mutant huntingtin RNA biology in disease pathogenesis^10–13^. First, expanded CAG repeats adopt secondary conformations and scaffold multivalent interactions that drive length-dependent phase separation and clustering in vitro^14^. Second, in human HD fibroblasts, mutant *HTT* mRNAs are retained in the nucleus, sequester splicing factors such as MBNL1, and induce splicing defects^15^. Third, comparison of two HD mouse models: one expressing a translatable *HTT* transgene (HD/100Q) and the other non-translatable variant (HD/100CAG), revealed that mutant *HTT* mRNA alone contributes to behavioural and transcriptional abnormalities, albeit less severely than the protein-expressing model^13^.

In this study, we leveraged a range of pharmacological treatments in YAC128-derived hippocampal cultures to probe the malleability of *HTT* nuclear RNA clusters. We observed that their assembly depends on ionic interactions and can be disrupted by nucleic acid intercalators, highlighting their plasticity. Moreover, we established that unperturbed transcription nucleates these structures and that blockage of canonical splicing disrupts *HTT* nuclear RNA clusters and increases cytoplasmic *HTT1a* levels. An in-depth study of huntingtin RNA metabolism, as well as the nature and identity of *HTT* RNA clusters, will enhance our understanding of HD pathogenesis and inform therapeutic development.

## Results

### Huntingtin nuclear RNA clusters arise early in the mouse development

Previous studies documented that human full-length huntingtin and the alternatively processed *HTT1a* mRNAs form sizable clusters in brain cell nuclei in YAC128 and BACHD-97Q-△N17 HD transgenic mouse models^8,9^. This phenomenon was observed at 2 and 8 months of age in YAC128 animals^8,9^ and at 1,3, 6 and 8 months of age in BACHD-97Q-△N17 mice^9^.

To elucidate the temporal onset of this process, we set out to understand when *HTT* nuclear RNA clusters first appear. We collected brain tissue of YAC128 mice at three different developmental stages: embryonic day E14.5 and postnatal stages P1 and P15, and subjected tissues to highly sensitive and specific RNAscope experiments. To understand whether huntingtin nuclear RNA clusters change in late adulthood, we included brain tissue from 12-month-old mice in the analysis.

**Fig. 1:**
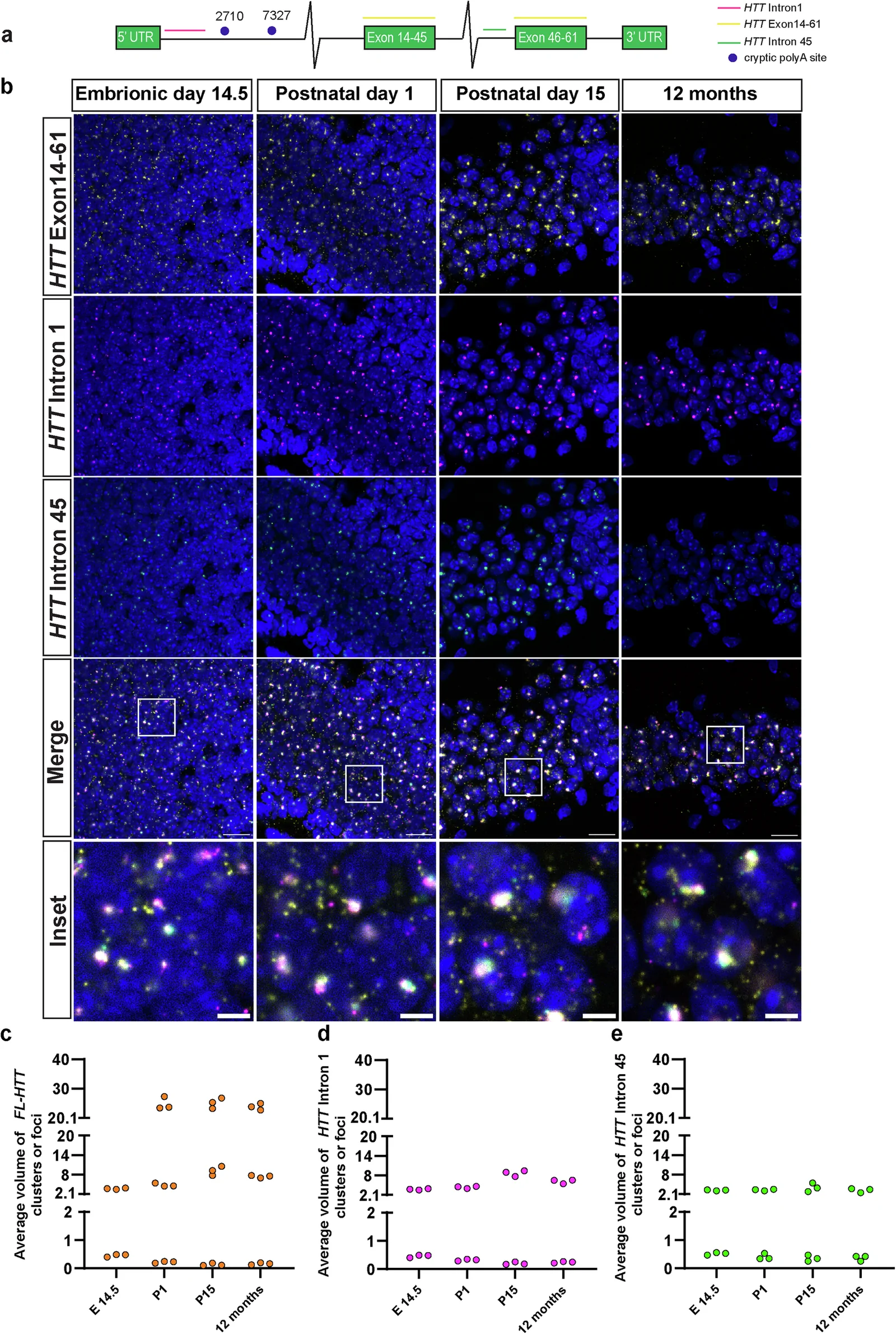
*HTT* nuclear RNA clusters arose early in the development of YAC128 mice. **a** Schematic showing the location of the RNAscope probes on the human *HTT* transcript: full-length *HTT* (*HTT*exon 14–61), *HTT1a* (*HTT* intron1) and pre-processed mRNA (*HTT* intron45). **b** *HTT* nuclear RNA clusters composed of full-length *HTT* (yellow) and *HTT1a* (magenta) were detected already at embryonic stage 14.5 in the medial pallium, the progenitor of the hippocampus. These clusters were also observed in postnatal brains 1 day or 15 days after birth, as well as in the aging animals at 12 months of age. Full-length *HTT* was also detected in the extranuclear space as single transcripts. The *HTT1a* mRNA was predominantly nuclear, although single mRNA molecules were observed in the extranuclear space. The *HTT* intro*n*45 (green) probe visualised non-spliced pre-mRNA, which co-stained *HTT* nuclear clusters. Quantification of the average volume of **c** FL-*HTT* RNA, **d** *HTT* intron1 or **e** *HTT* intron45 clusters or transcripts at different ages of YAC128 animals. Nuclei were stained with DAPI (blue). The wild-type control sections at the corresponding ages are shown in Supplementary Fig. 1c. Number of YAC128 animals used (*n* = 3). Scale bar is 20 μm in the main image and 5 μm in the cropped magnified image. Supporting data are provided in Supplementary Data 1.

We took advantage of previously designed probes to detect either human *HTT1a* (5′ sequences of intron 1) or FL-*HTT* (exons 14–61) (Fig. 1a). We used a novel probe to identify unprocessed *HTT* pre*-*mRNA (intron 45) (Fig. 1a) as this probe uncovered higher levels of pre-mRNA present in the clusters than previously appreciated using the *HTT*inton66 probe (Supplementary Fig. 1a).

YAC128 brain sections were hybridised with the three RNAscope probes, and the medial pallium, a precursor structure that gives rise to the hippocampus, was imaged in pre- and early post-natal animals (Supplementary Fig. 1b), and the hippocampus was imaged in the adult 12-month-old mice. The specificity of the human *HTT* probes was confirmed, as they gave no signal when hybridised to brain sections from wild-type mice (Supplementary Fig. 1c).

**Fig. 2:**
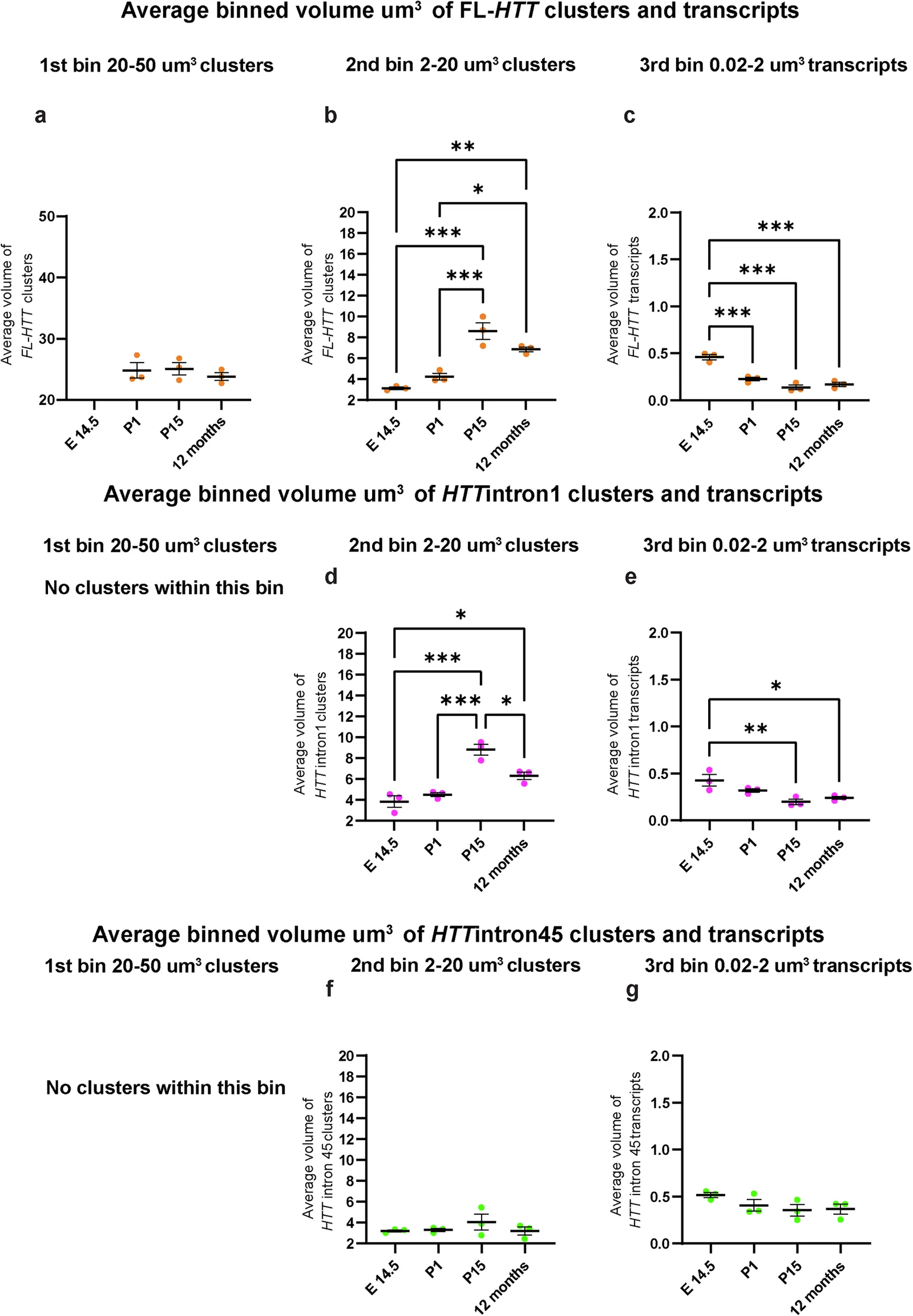
Volume of *HTT* nuclear RNA clusters was significantly changed at different developmental stages. Following quantification of the volume of *HTT* nuclear clusters and single mRNA molecules in the nucleus, results were binned into three separate categories to facilitate visual representation and statistical analysis. **a** Average volume of the FL*-HTT* clusters in the first bin remain unchanged at different developmental stages (P1 = 25 ± 1, P15 = 25 ± 1, 12-months = 24 ± 0.6). **b** FL*-HTT* clusters in the second bin differed significantly in average volume between different ages (E14.5 = 3.250 ± 0.06, P1 = 4.80 ± 0.3, P15 = 9.2 ± 0.8, 12-months = 7.4 ± 0.2). **c** The average volume of the single FL-*HTT* transcripts was also affected by development (E14.5 = 0.45 ± 0.03, P1 = 0.2 ± 0.02, P15 = 0.13 ± 0.02, 12-months = 0.16 ± 0.02). **d** The average volume of *HTT* intron1 clusters in the second bin was impacted markedly by age (E14.5 = 3.6 ± 0.5, P1 = 4.4 ± 0.2, P15 = 8.6 ± 0.5, 12-months = 6.0 ± 0.4). **e** Changes in the volume of the single *HTT1a* molecules mirrored that of FL-*HTT* (E14.5 = 0.4 ± 0.06, P1 = 0.3 ± 0.02, P15 = 0.2 ± 0.02, 12-months = 0.2 ± 0.02). **f** The average volume of *HTT* intron45 clusters in the second bin did not differ significantly between pre- and post-natal animals (E14.5 = 3.2 ± 0.09, P1 = 3.3 ± 0.01, P15 = 4.0 ± 0.8, 12-months = 3.3 ± 0.4). **g** The average volume of the single *HTT* intron45 transcripts remained unchanged across development (E14.5 = 0.51 ± 0.03, P1 = 0.4 ± 0.06, P15 = 0.35 ± 0.06, 12-months = 0.4 ± 0.05). FL-*HTT* (yellow), *HTT* intron1 (magenta), *HTT* intron45 (green), nuclei were stained with DAPI (blue). Number of YAC128 animals used (*n* = 3). Scale bar is 20 μm in the main image and 5 μm in the cropped magnified image. The Shapiro–Wilk test was applied to test data for normal distribution. Supporting data are provided in Supplementary Data 1. The significance of data was verified either by one-way ANOVA. Error bars = mean ± SEM, *P ≤ 0.033, **P ≤ 0.002, ***P ≤ 0.0002 and ****P ≤ 0.0001.

Using Fiji software^16^, we established a protocol to calculate volumes and numbers of nuclear clusters as well as nuclear and extranuclear single transcript molecules (Supplementary Fig. 2a). For quantification of *HTT* nuclear RNA clusters, we used an arbitrary cut-off of 2 µm^3^ to define an RNA cluster. This threshold was set based on quantification of the volume of mouse nuclear transcripts whose volume did not exceed 2 µm^3^. Single nuclear mRNA transcripts were described as puncta that ranged from 0.025 to 2 µm^3^ in volume. Single nuclear mRNAs and clusters were then binned into three separate categories based on their volume to facilitate visualisation and graphical representation as well as statistical analysis. The first bin contained large clusters between 20 and 50 µm^3^, the second bin contained smaller clusters between 2 and 20 µm^3^, whereas the third bin incorporated transcripts between 0.025 and 2 µm^3^. Figures 1 and 2 show animal-level averages for each of these three bins, whereas Supplementary Fig. 3 depicts the full distribution of all individual cluster and transcript measurements obtained across the analysed sections.

The FL-*HTT* probe revealed large RNA clusters detained in the cells of neuronal origin but not in the glial cells, which mainly contained single FL-*HTT* mRNAs (Supplementary Fig. 2b). Huntingtin nuclear RNA clusters, as well as single FL-*HTT* transcripts present in the nucleus and cytoplasm, were detected in embryonic, postnatal and adult YAC128 mice (Fig. 1b, c). The biggest FL-*HTT* clusters detected in the early postnatal and adult mice reached up to 45 µm^3^, whereas in mice at E14.5 these clusters were significantly smaller with the largest clusters approaching 17 µm^3^ in volume (Supplementary Fig. 3a). Interestingly, there was a marked increase in the average volume of the smaller FL-*HTT* clusters classified in the 2nd bin (2–20 µm^3^), between early postnatal age P1 and later postnatal stage P15, and then a sharp decrease in 12-month-old mice, suggesting a dynamic regulation of cluster size across developmental and aging stages (Fig. 2a, b). The average volume of single intranuclear FL-*HTT* transcripts also differed significantly between various developmental stages, with a significant decrease occurring between embryonic/P1 mice and animals transitioning to adulthood/ older mice at 12 months of age (Fig. 2c).

Strikingly, cytoplasmic FL-*HTT* mRNAs were detected exclusively as single puncta and never assembled into larger structures. Their volumes never exceeded 1 µm^3^ (Supplementary Fig. 3b) and were comparable across all ages examined, except in 12-month-old animals, which showed smaller average volumes (Supplementary Fig. 3c).

As described previously, the intron1 probe (*HTT* intron1) was present predominantly in the nucleus and co-localised with *FL*-*HTT* mRNA in the clusters^8^. *HTT* intron1 transcripts formed clusters that did not exceeded 20 µm^3^ (Fig. 1d and Supplementary Fig. 3d). On average, the volume of the *HTT* intron1 clusters classified in the 2nd bin was similar to the volume of the clusters formed by FL-*HTT* (Fig. 2d). Again, we observed a significant rise in the average volume of *HTT* intron1 clusters between postnatal age P1 and postnatal stage P15 and then a marked decrease in 12-month-old mice, which mirrored the trend showed by FL-*HTT* clusters, suggesting that these clusters are regulated together. Changes in the average volume of single *HTT*intron 1 intranuclear transcripts followed those of FL-*HTT,* with significant differences detected between embryonic stage and mice undergoing the transition to adulthood or older animals at 12 months (Fig. 2e).

Importantly, cytoplasmic *HTT1a* transcripts were detected in mice at all investigated stages. Quantification of the volume of extranuclear puncta revealed no significant differences between groups (Supplementary Fig. 3e, f). These results are in accordance with the *HTT1a* transcript being transported to the cytoplasm for translation to generate the HTT1a protein.

We previously reported that huntingtin nuclear RNA clusters were rarely identified by the intron66 probe^8^, suggesting they were enriched in the spliced *HTT* mRNA. In the current study, we used another intronic probe, *HTT* intron45 (Fig. 1a), to visualise unprocessed huntingtin mRNA. We observed nuclear clustering of *HTT* intron45 in mice at all developmental stages (Fig. 1b, e). Clusters formed by *HTT* intron45 were similar in average volume to those formed by *FL*-*HTT* or *HTT* intron1 transcripts for animals at E14.5 and P1, but their size remained unchanged later in adulthood (Fig. 2f and Supplementary Fig. 3g). Single intranuclear pre-mRNA *HTT* intron45 molecules showed comparable average volumes across different life stages of YAC128 animals (Fig. 2g). These data reveal a previously underappreciated presence of unprocessed pre-mRNA within the huntingtin nuclear RNA clusters.

**Fig. 3:**
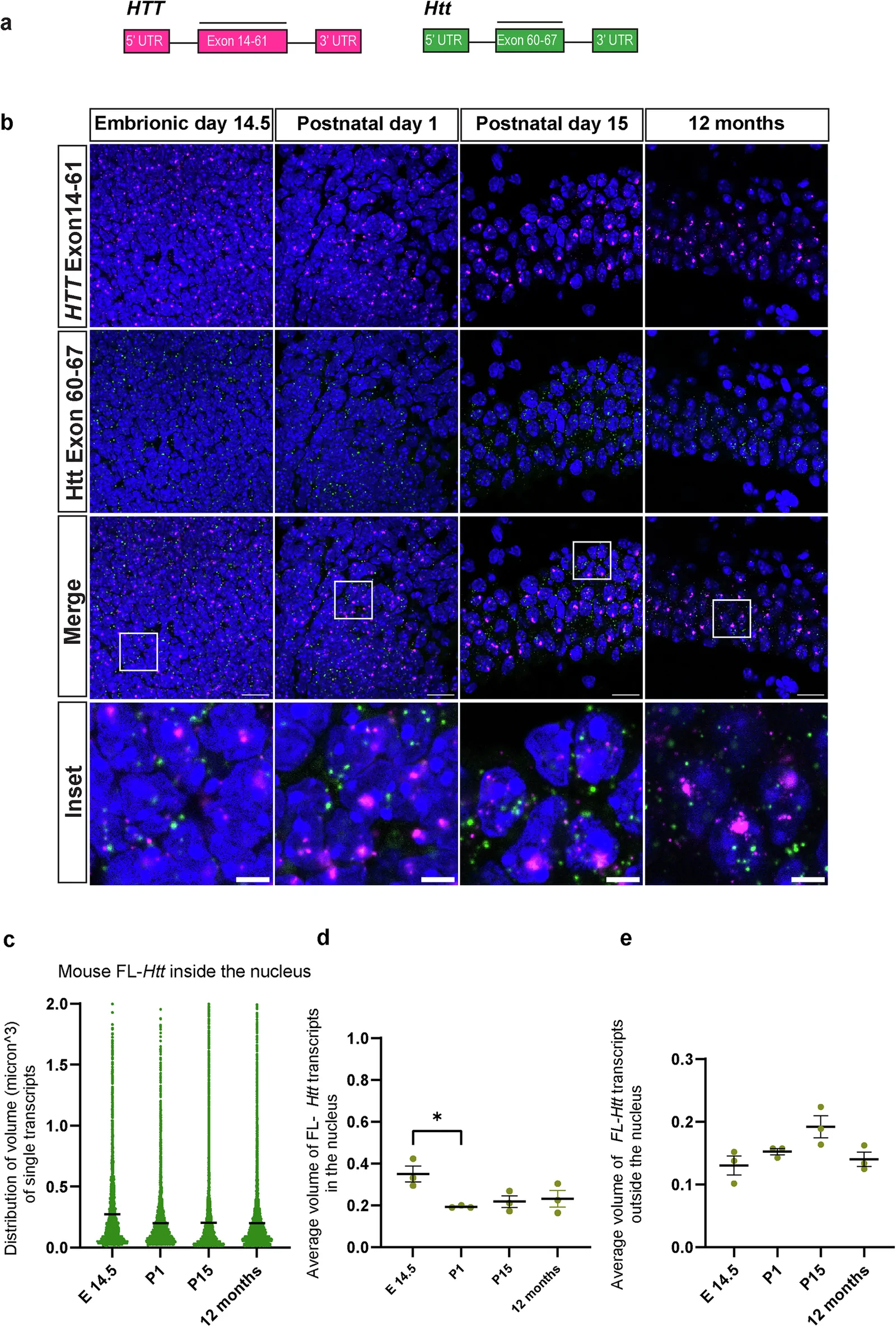
Human and mouse huntingtin mRNAs were retained in the nucleus, however only human *HTT* formed clusters. **a** Schematic location of the full-length human (*HTT* exon14–61) and mouse (*Htt* exon60–67) huntingtin RNAscope probes. **b** Full-length human *HTT* (magenta) was detected in large nuclear clusters and as single transcripts in the extranuclear space. Full-length mouse *Htt* (green) was mostly enriched in the nucleus, with single molecules found in the extranuclear space. **c** Distribution of the volume of FL-*Htt* transcripts inside the nucleus. Quantification of the averaged volume of mouse FL-*Htt* transcripts detected **d** inside (E14.5 = 0.35 ± 0.04, P1 = 0.2 ± 0.003, P15 = 0.2 ± 0.03, 12-months = 0.2 ± 0.04) or **e** outside of the nucleus at different ages of YAC128 mice (E14.5 = 0.13 ± 0.015, P1 = 0.15 ± 0.005, P15 = 0.19 ± 0.002, 12-months = 0.14 ± 0.01). Nuclei were stained with DAPI (blue). Number of YAC128 animals used (*n* = 3). Scale bar is 20 μm in the main image and 5 μm in the cropped magnified image. The Shapiro–Wilk test was applied to test data for normal distribution, and one-way ANOVA was subsequently used to verify the significance of data for the transcripts inside the nucleus, whereas the Kruskal–Wallis test was applied to test the differences in the transcripts outside the nucleus. Supporting data are provided in Supplementary Data 1 and 2. Error bars = mean ± SEM, *P ≤ 0.033, ***P ≤ 0.0002 and ****P ≤ 0.0001.

Interestingly, volumetric analysis of mouse wild-type FL*-Htt* in the nucleus revealed that these transcripts comprised a homogenous population with mRNA molecules not surpassing 2 µm^3^ (Fig. 3a–c). On average, the volume of nuclear FL*-Htt* was significantly elevated in the embryos and showed no variations postnatally (Fig. 3d). Moreover, the average volume of extranuclear FL*-Htt* molecules was unchanged across developmental stages and in late adulthood (Fig. 3e).

As published earlier^8^, nuclear retention and cluster formation was specific to the FL*-HTT/HTT1a* transcripts as housekeeping mRNAs, including *Ubc*, *Ppib* and *Polar2a*, were primarily localised in the cytoplasm and detected at all ages investigated (Supplementary Fig. 4). Hybridisation with a set of bacterial probes that served as a negative control rendered no staining (Supplementary Fig. 5).

### Huntingtin RNA clusters associate with RNA splicing machinery but do not accumulate nearby well-characterised nuclear bodies

**Fig. 4:**
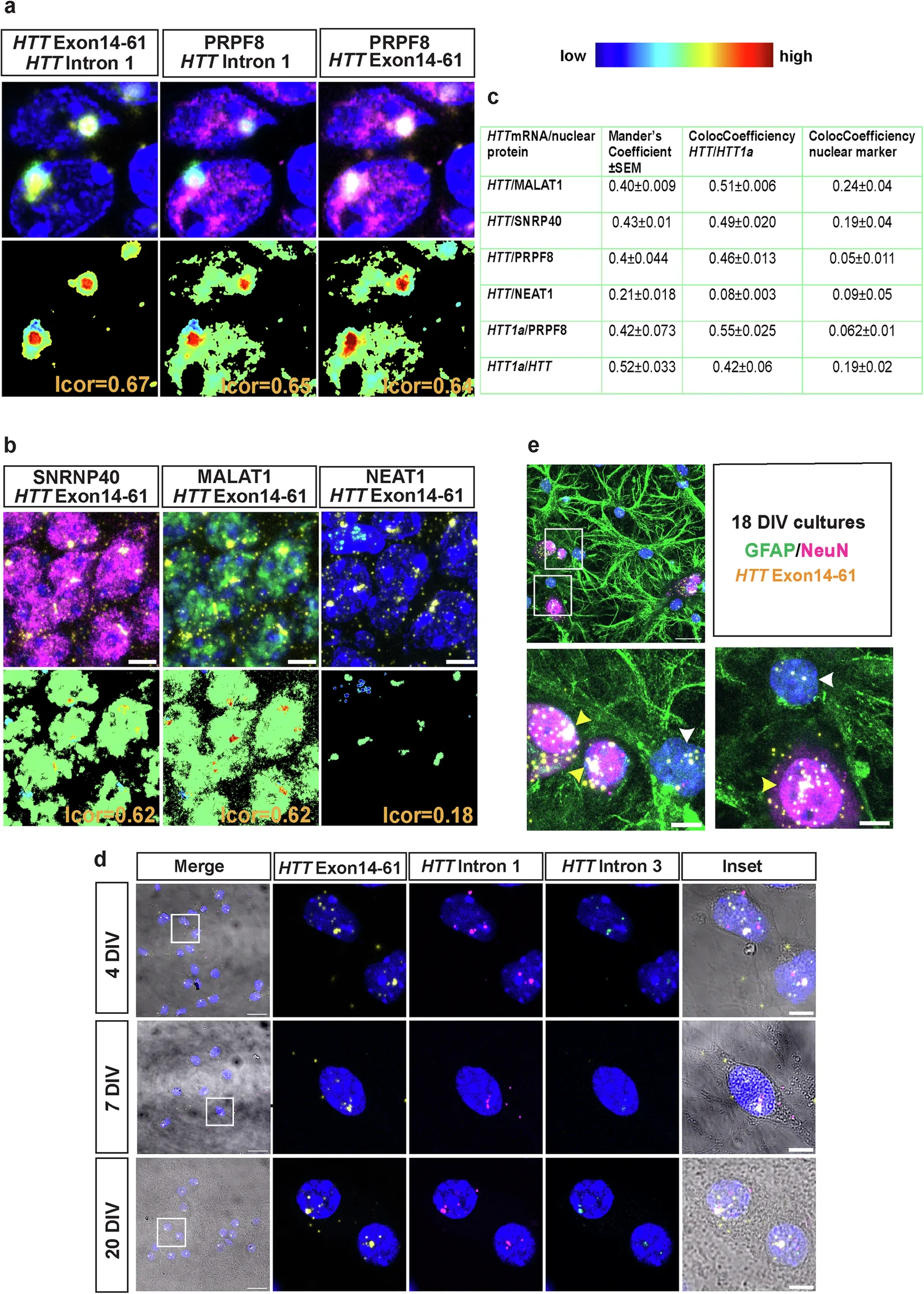
*HTT* nuclear RNA clusters colocalised with splicing machinery and were present in developing and mature hippocampal cultured neurons. **a** Representative images depicting that FL-*HTT* (yellow) and *HTT1a* (green) mRNAs colocalised within the nucleus. Additionally, both transcripts colocalised with the marker of the splicing machinery (PRPF8, magenta). **b** *HTT* clusters (yellow) were also associated with another essential splicing protein SNRNP40 (magenta), and a marker of the splicing speckles lncRNA* MALAT1* (green), but did not colocalise with lncRNA* NEAT1*(green), a component of paraspeckles. Icor represents the ‘index of correlation’ and indicates the extent of colocalization. Analysis was performed using the ImageJ plug-in colocalisation colormap. **c** A table demonstrating Mander’s correlation coefficient as well as colocalisation coefficiency 1 and colocalisation coefficiency 2 of images shown in (**a**, **b**). All the values were computed using Zeiss Blue 3.9 software. **d** RNAscope images showed that *HTT* nuclear RNA clusters formed as early as 4 DIV in cultured hippocampal cells and persisted when cells reached maturity (20 DIV). Full-length human *HTT* exon14–61 (yellow), *HTT* intron1 (magenta), *HTT* intron3 (green). Brightfield filter was added to better discriminate neuronal morphology. **e** Combined RNAscope-immunofluorescence experiments revealed that *HTT* nuclear RNA clusters were predominantly present in the cells of neuronal origin (indicated with yellow arrowheads), whereas in GFAP-positive astrocytes, single *HTT* mRNA molecules were detected (white arrowhead). GFAP, astrocytic marker (green), NeuN stained neuronal nuclei (magenta), FL-*HTT* (yellow), DAPI, nuclear stain (blue). Number of individual cultures *n* = 3, number of transgenic mouse pups per culture imaged *n* = 2. Scale bar is 20 μm in the main image and 5 μm in the cropped magnified image. Supporting data are provided in Supplementary Data 1.

The nuclear interior is organised around chromatin into specific, microscopically visible nuclear substructures, called nuclear bodies, which play a distinct role in RNA metabolism^17^. To understand the molecular identity of *HTT* nuclear RNA clusters, we combined RNAscope and immunofluorescence (RNAscope-IF) and stained YAC128 brain sections at 2 months for markers of well-identified nuclear bodies. Given that these structures contain *HTT* intron45 and are adjacent to transcription sites^9^, we hypothesised they could be localised near the splicing machinery. Several proteins captured by mutant *HTT* RNA belong to the spliceosome pathway, including PRPF8 and SNRNP40^15,18^, that also interact with in vitro transcribed *HTT* exon1^18^. RNAscope-IF revealed robust colocalization of PRPF8 with FL*-HTT/HTT* intron1-rich clusters (Fig. 4a and Supplementary Fig. 6a). Subsequent analysis, using the Colocalisation colormap plug-in in ImageJ software^19^, confirmed a strong spatial association of PRPF8 with *HTT* intron1 (Icor = 0.65) and FL-*HTT* (Icor = 0.64) nuclear clusters, comparable in strength to the binding between *HTT* intron1 and FL*-HTT* (Fig. 4a, Icor = 0.67). To strengthen the evidence that *HTT* nuclear clusters are associated with the splicing machinery, we examined another spliceosomal component, SNRNP40. PRPF8 and SNRNP40 are known to functionally interact, and both proteins are essential for efficient pre-mRNA splicing^18^. Consistently, *HTT* nuclear clusters showed strong spatial association with SNRNP40 (Fig. 4b and Supplementary Fig. 6b, Icor = 0.62), supporting the idea that these structures are positioned within sites enriched in RNA processing factors.

We next investigated whether *HTT* nuclear clusters interact with long non-coding RNAs: *MALAT1*, a component of nuclear speckles involved in splicing regulation^20,21^, and* NEAT1*, the architectural RNA of paraspeckles implicated in gene regulation^22^. *HTT* nuclear clusters displayed clear association with *MALAT1* (Fig. 4b and Supplementary Fig. 6b, Icor = 0.62) but not with* NEAT1*(Fig. 4b and Supplementary Fig. 6c, Icor =  0.18), suggesting that *HTT* clusters preferentially localise to splicing-related nuclear domains rather than paraspeckles. To complement the spatial analysis, we quantified Manders’ colocalization coefficient (MCC), as well as colocalisation coefficiency1 and colocalisation coefficiency2 (Fig. 4c). MCC measures the fraction of signal that coincides between two channels^23,24^. The MCC values obtained (~0.40–0.50) indicate a moderate degree of overlap between *HTT* nuclear clusters and the nuclear markers analysed. Because MCC reflects global signal overlap and does not capture spatial distribution, these values were interpreted alongside colocalisation colormap analysis, which provides spatially resolved information. The colocalization coefficiency corresponding to the *HTT* nuclear cluster channel (ColocCoefficient1) ranged from ~0.40–0.50, indicating that approximately half of the cluster signal overlaps with the analysed nuclear markers. In contrast, the reciprocal coefficiency for the nuclear protein channel (ColocCoefficient2) was substantially lower (~0.20–0.05). This asymmetry indicates that while a considerable fraction of the cluster signal coincides with the nuclear markers, only a small proportion of the total nuclear protein signal resides within clusters. These values are consistent with punctate structures embedded within a broadly distributed nuclear signal and further support the spatial association detected by the colocalisation colormap analysis. Next, we wondered whether *HTT* nuclear RNA structures were enriched within other nuclear markers and organelles such as nucleolus (rRNA45S), Cajal bodies (coilin), paraspeckles (SFPQ), open state chromatin (H3K9ac), RNA nuclear export (p84) or different RNA-binding proteins (TDP-43, hnRNPF, hnRNPK). Analysis of the experimental data demonstrated that none of the tested markers colocalised with *HTT* nuclear RNA clusters (Supplementary Fig. 7).

These data complement previous reports that several splicing factors, including PRPF8 and SNRNP40, can be trapped by mutant *HTT* RNA and further support the idea that these interactions might play a role in the alternative processing and production of *HTT1a*.

### Inhibitor of electrovalent bond disrupts huntingtin nuclear RNA clusters

Next, we asked whether *HTT* nuclear RNA clusters constitute malleable structures that exhibit dynamic behaviour or whether they are static. To probe this question, we took advantage of mixed primary hippocampal cultured cells derived from YAC128 newborn pups. First, we established that these cells show *HTT* nuclear RNA clusters. We performed RNAscope using three sets of probes against human *HTT1a*, FL*-HTT* or unprocessed *HTT* pre*-*mRNA (intron3). We interrogated developing hippocampal cells at ‘days in vitro’ (DIV) 4, cells at DIV 7 and fully matured cultures at DIV 18–20. RNAscope analysis revealed the presence of *HTT* nuclear RNA clusters already at DIV 4 that persisted at least up until DIV 20 (Fig. 4d). Moreover, RNAscope-IF confirmed that *HTT* nuclear RNA clusters were localised to neurons and not glia (Fig. 4e). Thus, mixed primary hippocampal cells constitute a reliable model to study the plasticity of clusters.

**Fig. 5:**
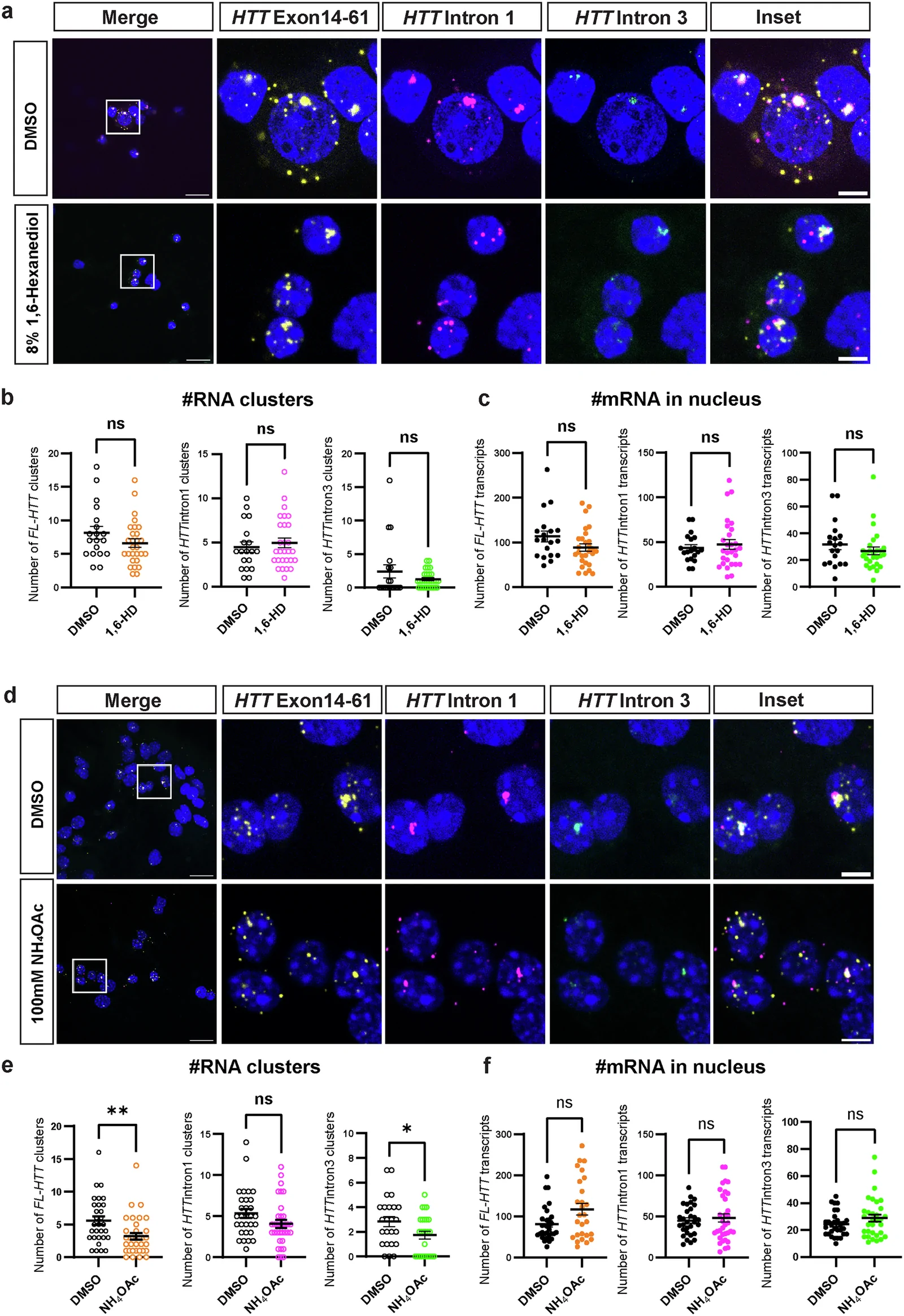
Liquid-liquid phase separation did not drive *HTT* nuclear RNA cluster formation, but these structures were disrupted by an agent that perturbs ionic interactions. **a** Hippocampal cultures at 18–20 DIV were treated with 8% 1,6-hexanediol or DMSO for 5 min. 5% 1,6-hexanediol and 8% 2,5-hexanediol were also tested, and the images are shown in Supplementary Fig. 8. Quantification of **b** the number of *HTT* RNA clusters revealed no major changes (FL-*HTT*: 1,6-HD = 6.6 ± 0.6, DMSO = 8.2 ± 0.9; *HTT* intron1: 1,6-HD = 4.9 ± 0.5, DMSO = 4.5 ± 0.6; *HTT*intron 3: 1,6-HD = 1.2 ± 0.2, DMSO = 2.4 ± 1. **c** The number of puncta in the nucleus remained unaffected (FL-*HTT*: 1,6-HD = 88 ± 8, DMSO = 113 ± 12; *HTT*intron 1: 1,6-HD = 47 ± 5, DMSO = 43 ± 3; *HTT* intron3: 1,6-HD = 27 ± 3, DMSO = 32 ± 4). **d** Hippocampal cultures at 18 DIV were treated with 100 mM ammonium acetate for 10 min. **e** Quantification of the number of *HTT* RNA clusters showed significant differences in the amount of FL-*HTT* and *HTT* intron3 nuclear clusters but not *HTT* intron1 nuclear clusters, although a trend toward lower number was observed (FL-*HTT*: NH_4_OAc = 3.2 ± 0.5, DMSO = 5.6 ± 0.6; *HTT* intron1: NH_4_OAc = 4 ± 0.5, DMSO = 5.3 ± 0.5; *HTT* intron3: NH_4_OAc = 1.7 ± 0.3, DMSO = 2.8 ± 0.4). **f** The number of single mRNA molecules in the nucleus remain unchanged (FL-*HTT*: NH_4_OAc = 117 ± 14, DMSO = 81 ± 7; *HTT* intron1: NH4OAc = 48 ± 5, DMSO = 45 ± 3; *HTT* intron3: NH_4_OAc = 29 ± 3, DMSO = 24 ± 9). FL-*HTT* (yellow), *HTT* intron1 (magenta), *HTT* intron3 (green), nuclei were stained with DAPI (blue). Number of individual cultures *n* = 3, number of transgenic mouse pups per culture imaged *n* = 2. Each data point represents a field of view (ROI) imaged within a culture (~10–15 cells per ROI). Scale bar is 20 μm in the main image and 5 μm in the cropped magnified image. The Shapiro–Wilk test was applied to test data for normal distribution, and the Mann–Whitney test was subsequently used to verify the significance of the data. Error bars = mean ± SEM, *P ≤ 0.033, **P ≤ 0.002, ***P ≤ 0.0002 and ****P ≤ 0.0001. Supporting data are provided in Supplementary Data 1.

Exogenously expressed repeat-containing RNA has been reported to undergo phase separation and exhibit liquid-like properties^14^. To probe whether *HTT* nuclear RNA clusters display characteristics of liquid–liquid phase separated condensates (LLPS), we took advantage of 1,6-hexanediol (Fig. 5a and Supplementary Fig. 8) and 2,5-hexanediol (Supplementary Fig. 8), agents known to hinder weak hydrophobic interactions that hold LLPS separated molecules together. Biological structures that rely upon LLPS are sensitive to 1,6-hexanediol, but less sensitive to less potent 2,5-hexanediol^17^. A 5-min application of 8% 1,6-hexanediol to hippocampal cells did not exert any effect on the number of *HTT* nuclear RNA clusters (Fig. 5b). The number of intranuclear transcripts remained unaltered (Fig. 5c), as did the levels of cytoplasmic huntingtin mRNA molecules (Supplementary Fig. 9b). We concluded that LLPS was not a major driving force behind *HTT* nuclear RNA clusters formation.

To assess whether ionic interactions affect RNA cluster stability, we applied ammonium acetate (100 mM), a cell-permeable agent that does not disturb intracellular pH^14,25^. Surprisingly, a 10-min treatment was enough to prompt a notable decrease in the number of *HTT* nuclear RNA clusters. This decrease was significant for the FL*-HTT* and *HTT* intron3 and showed a downward trend that did not reach significance for *HTT* intron1 (Fig. 5d, e). Interestingly, the volume of *HTT* nuclear RNA clusters was also diminished upon NH_4_OAc treatment (Supplementary Fig. 9c), suggesting that the remaining few clusters either persist as dwarfed entities or will be dynamically dis/re-assembled later. The total number of single intranuclear mRNA huntingtin molecules was unchanged (Fig. 5f), whereas the number of cytoplasmic transcripts varied only for *HTT1a*, showing a significant drop (Supplementary Fig. 9d). These results suggest that *HTT* nuclear RNA cluster assembly is partly regulated by ionic interactions, which in biological systems may be facilitated by RBPs that help neutralise the negative charge of RNA molecules.

### Agents that intercalate into nucleic acids and block transcription induce disassembly of huntingtin nuclear RNA clusters

**Fig. 6:**
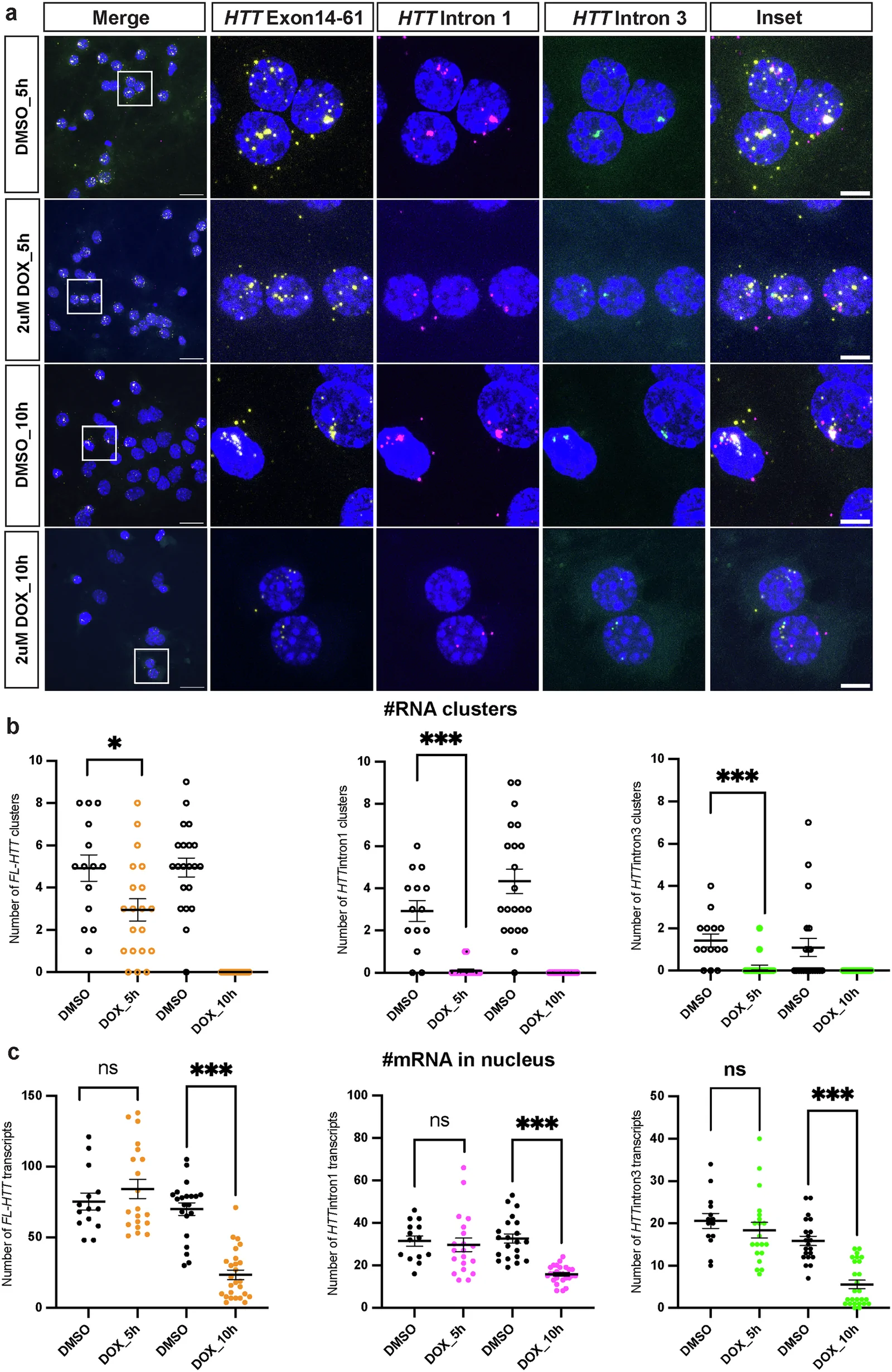
HTT nuclear RNA clusters disintegrate in response to doxorubicin. **a** Two micromolar doxorubicin was applied to hippocampal cultures at 18–20 DIV either for 5 or 10 h. **b** Doxorubicin diminished the number of *HTT* nuclear RNA clusters after 5 h and completely abrogated their numbers after the 10-h incubation (5 h: FL-*HTT*: Dox = 3 ± 0.5, DMSO = 5 ± 0.6; *HTT* intron1: Dox = 0.1 ± 0.07, DMSO = 3 ± 0.5, *HTT* intron3 Dox = 0.15 ± 0.1 DMSO = 1.4 ± 0.3; 10 h: FL-*HTT*: Dox = 0.0 ± 0.0, DMSO = 5 ± 0.45; *HTT* intron1: Dox = 0.0 ± 0.0, DMSO = 4.3 ± 0.6, *HTT* intron3 Dox = 0.0 ± 0.0 DMSO = 1 ± 0.4). **c** The number of single mRNA molecules inside the nucleus showed an initial trend toward an increase after 5 h for FL-*HTT*, followed by significant downregulation after 10 h (5 h: FL-*HTT*: Dox = 84 ± 6, DMSO = 75 ± 6; 10 h FL-*HTT*: Dox = 24 ± 3, DMSO = 70 ± 4). The levels of intranuclear *HTT* intron1 and *HTT* intron3 transcripts decreased progressively (5 h: *HTT* intron1: Dox = 29.6 ± 3 DMSO = 31 ± 2, *HTT* intron3: Dox = 18 ± 2 DMSO = 20 ± 2; 10 h: *HTT* intron1:Dox = 16 ± 0.7 DMSO = 33 ± 2, *HTT* intron3: Dox = 6 ± 1 DMSO = 16 ± 1). FL-*HTT* (yellow), *HTT* intron1 (magenta), *HTT* intron3 (green), nuclei were stained with DAPI (blue). Number of individual cultures *n* = 3, number of transgenic mouse pups per culture imaged *n* = 2. Each data point represents a field of view (ROI) imaged within a culture (~10–15 cells per ROI). Scale bar is 20  μm in the main image and 5 μm in the cropped magnified image. Shapiro–Wilk test was applied to test data for normal distribution and either Mann–Whitney (5-h treatment: number of clusters for *HTT* intron1 and *HTT* intron3; number of nuclear transcripts for FL-*HTT* and *HTT* intron1; 10-h treatment: number of clusters for all three probes; number of nuclear transcripts for FL-*HTT*, number of nuclear transcripts for *HTT* intron3) or unpaired two-tailed t-test (5-h treatment: number of clusters for FL-*HTT* and number of nuclear transcripts for *HTT* intron3; 10-h treatment: number of nuclear transcripts for *HTT* intron1) was subsequently used to verify the significance of the data. Supporting data are provided in Supplementary Data 1. Error bars = mean ± SEM, *P ≤ 0.033, **P ≤ 0.002, ***P ≤ 0.0002 and ****P ≤ 0.0001.

Next, we tested whether base-pairing interactions play a role in maintaining *HTT* RNA cluster integrity. We applied doxorubicin (2 µM), a nucleic acid intercalator, which substantially reduced the number and total volume of clusters per cell in a progressive and time-dependent manner (Fig. 6a, b and Supplementary Fig. 9e). Importantly, the number of single nuclear mRNA transcripts was already diminished for all three probes, 5 h post-treatment and continued to decrease further (Fig. 6c). Cytoplasmic FL-*HTT* transcripts increased 5 h after doxorubicin application to then decrease significantly 10 h after drug treatment, *HTT1a* showed a downward time-dependent tendency (Supplementary Fig. 9f). In summary, these data suggest that intermolecular base-paring interactions contribute to the stability of *HTT* nuclear RNA clusters either by promoting an intact transcription process accompanied by efficient topoisomerase activity or by preventing the efflux of a subclass of specific RBPs^26^.

**Fig. 7:**
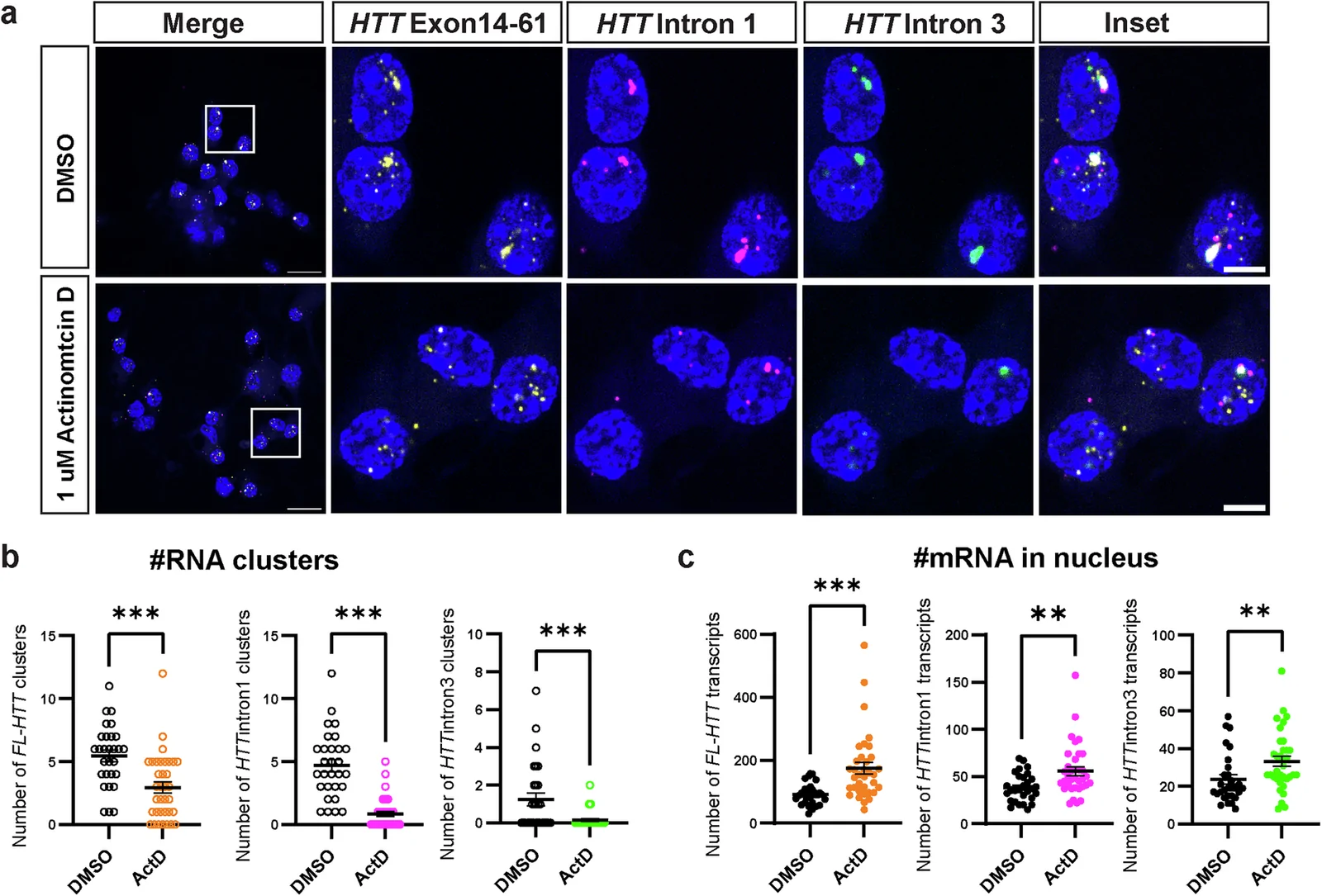
*HTT* nuclear RNA clusters were sensitive to transcription inhibition. **a** Hippocampal cultures at 18–20 DIV were treated with the commonly used transcription blocker 1 µM actinomycin D (ActD) for 3 h. **b** The inhibitor prevented huntingtin clustering and significantly downregulated the number of *HTT* nuclear RNA clusters (FL-*HTT*: ActD = 3 ± 0.4, DMSO = 5.5 ± 0.45; *HTT* intron1: ActD = 0.9 ± 0.2, DMSO = 4.7 ± 0.5, *HTT* intron3: ActD = 0.14 ± 0.07, DMSO = 1.2 ± 0.3). **c** The number of single mRNA molecules inside the nucleus was also notably affected (FL-*HTT*: ActD = 175 ± 2, DMSO = 91 ± 6, *HTT* intron1: ActD = 56 ± 5, DMSO = 38 ± 3, *HTT*intron 3: ActD = 33 ± 3, DMSO = 24 ± 2). FL-*HTT* (yellow), *HTT* intron1 (magenta), *HTT* intron3 (green), nuclei were stained with DAPI (blue). Number of individual cultures *n* = 3, number of transgenic mouse pups per culture imaged *n* = 2. Each data point represents a field of view (ROI) imaged within a culture (~10–15 cells per ROI). Scale bar is 20  μm in the main image and 5 μm in the cropped magnified image. The Shapiro–Wilk test was applied to test data for normal distribution, and the Mann–Whitney test was subsequently used to verify the significance of data. Supporting data are provided in Supplementary Data 1. Error bars = mean ± SEM, *P ≤ 0.033, **P ≤ 0.002, ***P ≤ 0.0002 and ****P ≤ 0.0001.

Given that *HTT* nuclear RNA clusters localise near transcription sites (as revealed by colocalization of FL-*HTT/HTT1a* clusters with *HTT* intron45), we explored the hypothesis that intact transcription might have an impact on the integrity of *HTT* nuclear RNA clusters. We first applied the commonly used pan-transcriptional inhibitor Actinomycin D (ActD, 1 µM), which alleviated not only the number of *HTT* nuclear RNA clusters (Fig. 7a, b) but also their volume (Supplementary Fig. 9g). In parallel, ActD raised the number of mRNA molecules inside the nucleus (Fig. 7c) as well as the levels of cytoplasmic mRNA puncta (Supplementary Fig. 9h).

### Inhibition of transcriptional elongation and pre-mRNA splicing promote dissolution of huntingtin nuclear RNA clusters

**Fig. 8:**
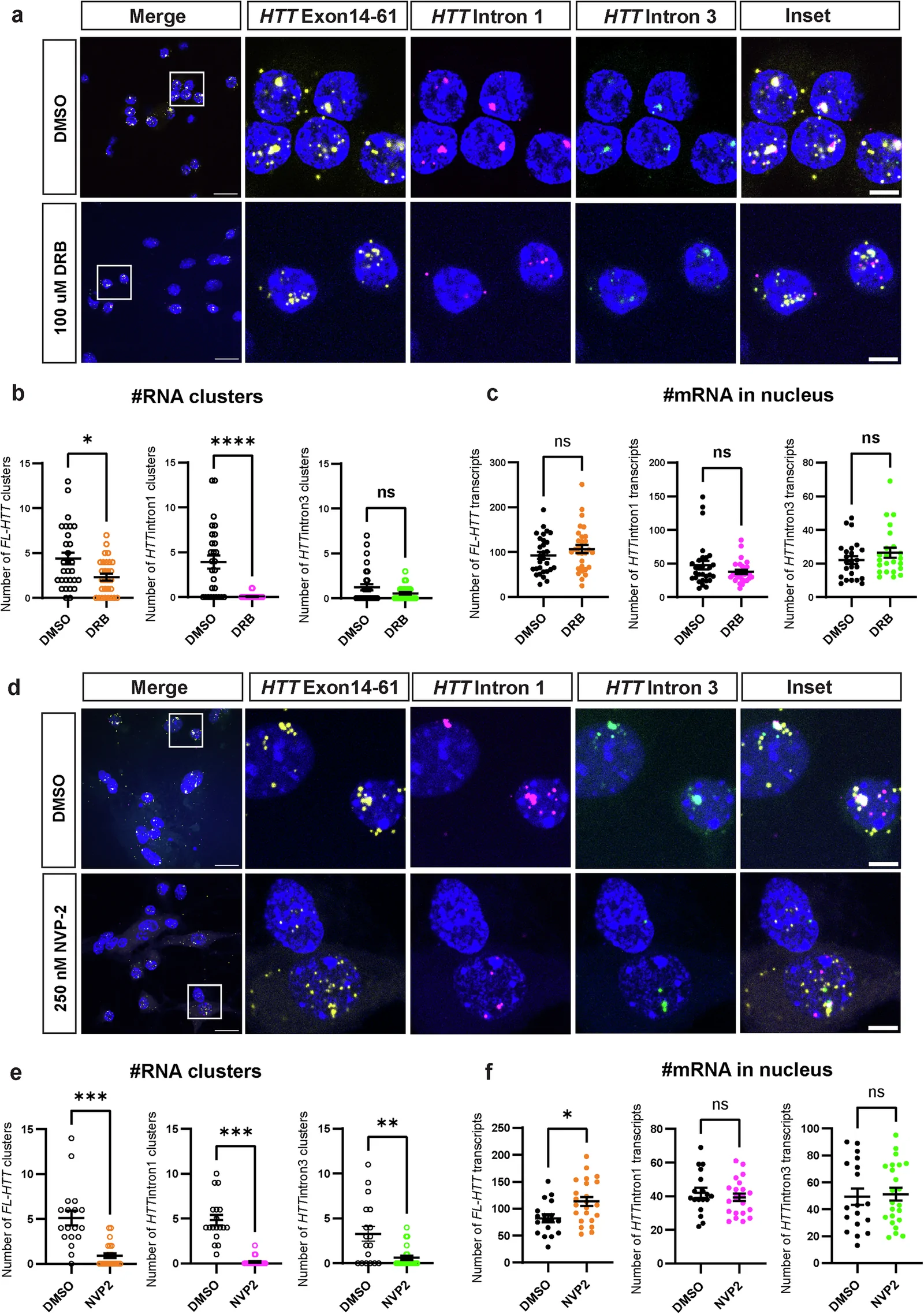
Inhibition of mRNA synthesis led to the disruption of *HTT* nuclear RNA clusters. Hippocampal cultures at 18–20 DIV were treated with transcription elongation inhibitors: **a** 250 nM NVP2 or **d** 100 µM DRB for 3 h. **b**, **e** Quantification analysis revealed that these pharmacological agents successfully dissolved *HTT* nuclear RNA clusters (DRB:FL-*HTT*:DRB = 2.3 ± 0.4, DMSO = 4.4 ± 0.6; *HTT* intron1 :DRB = 0.07 ± 0.05, DMSO = 4 ± 0.7; *HTT* intron3: DRB = 0.5 ± 0.1, DMSO = 1.2 ± 0.3; NVP-2: FL-*HTT*: NVP-2:0.9 ± 0.2 DMSO = 5 ± 0.8, *HTT* intron1: NVP-2 = 0.17 ± 0.1, DMSO = 4.8 ± 0.6; *HTT* intron3: NVP-2 = 0.6 ± 0.2, DMSO = 3.3 ± 0.8). **c** The number of FL-*HTT*, *HTT* inton1 or *HTT* intron3 transcripts in the nucleus remained unchanged upon DBR treatment (FL-*HTT*: DRB = 107 ± 9, DMSO = 93 ± 8; *HTT* intron1: DRB = 38 ± 3, DMSO = 48 ± 6, *HTT* intron3: DRB = 26 ± 3, DMSO = 22 ± 2). **f** NVP-2 had a significant effect on the number of FL-*HTT* molecules inside the nucleus without affecting numbers of either *HTT* intron1 or *HTT* intron3 transcripts (NVP-2: FL-*HTT*: NVP-2 = 113 ± 8, DMSO = 82 ± 7, *HTT* intron1: NVP-2 = 39 ± 2, DMSO = 42 ± 3; *HTT* intron3: NVP-2 = 51 ± 8, DMSO = 49 ± 6). FL-*HTT* (yellow), *HTT* intron1 (magenta), *HTT* intron3 (green), nuclei were stained with DAPI (blue). Number of individual cultures *n* = 3, number of transgenic mouse pups per culture imaged *n* = 2. Each data point represents a field of view (ROI) imaged within a culture (~10–15 cells per ROI). Scale bar is 20 μm in the main image and 5 μm in the cropped magnified image. The Shapiro–Wilk test was applied to test data for normal distribution, and either Mann–Whitney (DRB and NVP-2 treatments: number of clusters for all three probes) or unpaired two-tailed t-test (NVP-2 treatment: number of transcripts in the nucleus for all three probes; DRB: number of transcripts in the nucleus for FL-*HTT*) was subsequently used to verify the significance of data. Supporting data are provided in Supplementary Data. Error bars = mean ± SEM, *P ≤ 0.033, **P ≤ 0.002, ***P ≤ 0.0002 and ****P ≤ 0.0001.

To corroborate that *HTT* nuclear RNA clusters are sensitive to inhibition of mRNA transcription and avoid off-target effects of ActD, that include DNA-intercalation, we treated hippocampal cells either with 5,6-dichloro-1-β-D-ribofuranosylbenzimidazole (DRB, 100 μM) or with NVP-2 (250 nM). Both drugs inhibit transcription elongation and block CDK9-dependent RNA Pol II activation, with DRB having an additional negative effect on casein kinase II^27,28^. Like ActD, NVP-2 and DRB caused a significant drop in the number of *HTT* nuclear RNA clusters (Fig. 8a, b, d, e).

Strikingly, the total volume of FL-*HTT* nuclear RNA clusters was notably affected by both inhibitors, whereas *HTT* intron1 clusters were severely impacted only by NVP-2, an effect that might be attributed to the greater potency of NVP-2 (Supplementary Fig. 10a, c). Interestingly, only NVP-2 produced a substantial increase in the levels of intranuclear FL-*HTT* mRNAs, without affecting the number of *HTT* intron1 or *HTT* intron3 mRNAs (Fig. 8c, f).

None of the drugs significantly impacted the number of cytoplasmic transcripts (Supplementary Fig. 10b, d). This set of data underscores the importance of effective transcriptional elongation in the production of *HTT* nuclear RNA clusters.

**Fig. 9:**
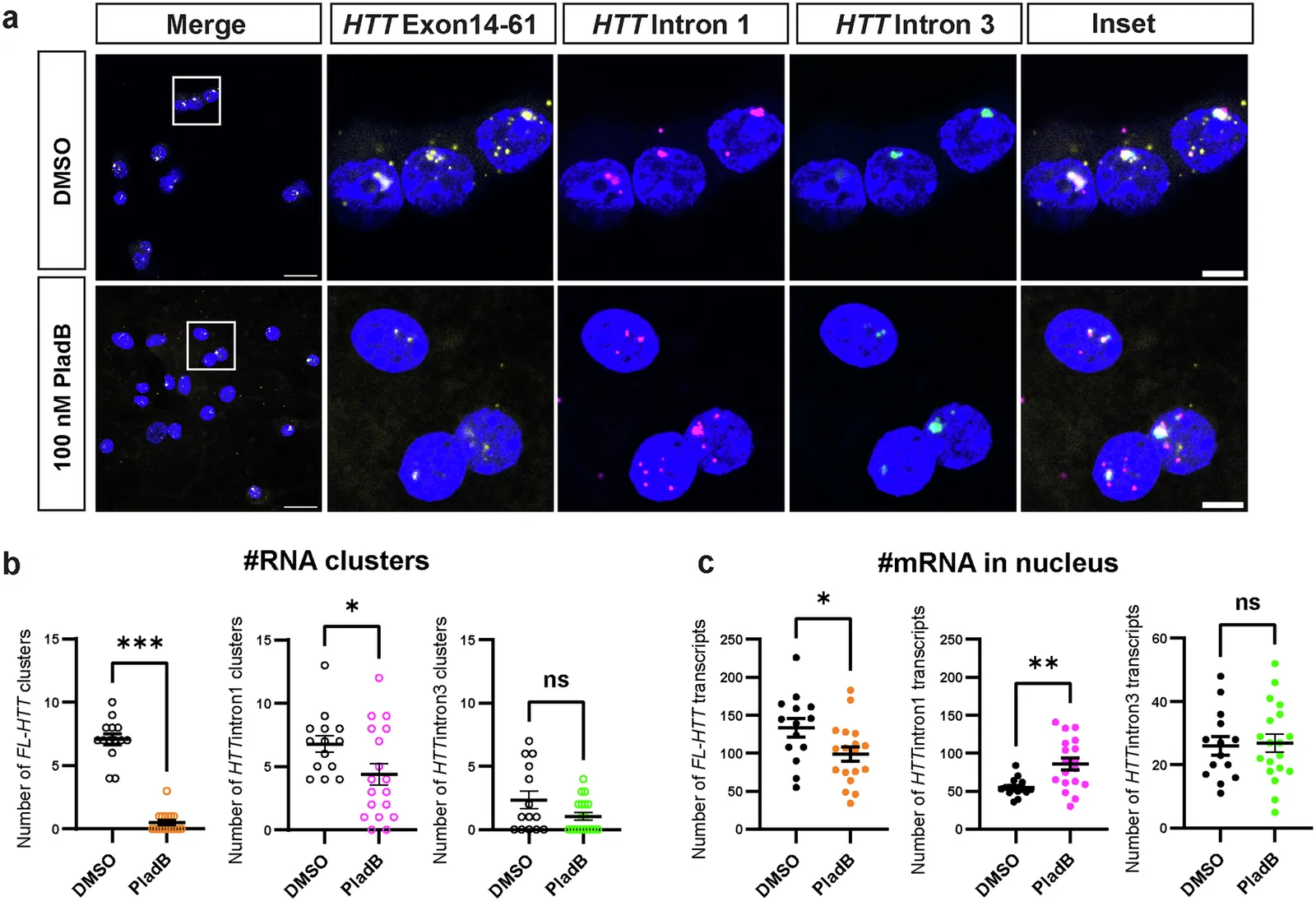
Inhibition of pre-mRNA splicing blocked formation of *HTT* nuclear RNA clusters and promoted accumulation of *HTT* intron1 mRNAs in the nucleus. **a** One hundred nanomolar of PladB was applied to 18–20 DIV hippocampal cells for 4 h. **b** The splicing inhibitor prompted a significant decrease in the number of *HTT* nuclear RNA clusters FL-*HTT*: PladB = 0.5 ± 0.2, DMSO = 7 ± 0.4; *HTT* intron1: PladB = 4.4 ± 0.8, DMSO = 6.8 ± 0.6, *HTT* intron3: PladB = 1.0 ± 0.3, DMSO = 2.3 ± 0.7), **c** PladB notably diminished the number of canonically spliced FL-*HTT* and significantly increased the amount of alternatively processed *HTT1a* (FL-*HTT*: PladB = 99 ± 9, DMSO = 133 ± 12, *HTT* intron1: PladB = 86 ± 8, DMSO = 55 ± 3, *HTT* intron3: PladB = 27 ± 3, DMSO = 26 ± 3). FL-*HTT* (yellow), *HTT* intron1 (magenta), *HTT* intron3 (green), nuclei were stained with DAPI (blue). Number of individual cultures *n* = 3, number of transgenic mouse pups per culture imaged *n* = 2. Each data point represents a field of view (ROI) imaged within a culture (~10–15 cells per ROI). Scale bar is 20 μm in the main image and 5 μm in the cropped magnified image. The Shapiro–Wilk test was applied to test data for normal distribution, and either Mann–Whitney (number of clusters for *HTT* intron1 and *HTT* intron3) or unpaired two-tailed t-test (number of clusters for FL-*HTT*, number of transcripts in the nucleus for all three probes) was subsequently used to verify the significance of data. Supporting data are provided in Supplementary Data 1. Error bars = mean ± SEM, *P ≤ 0.033, **P ≤ 0.002, ***P ≤ 0.0002 and ****P ≤ 0.0001.

Our data, together with those of others^18^, implicate the interaction of mutant *HTT* with components of the splicing machinery. Thus, we sought to understand whether pre-mRNA splicing plays a role in cluster formation. We used Pladienolide B (PladB, 100 nM), that targets the SF3B1 subunit in the U2 snRNP complex and inhibits early stages of the splicing reaction^28^. In concert with its effect on canonical splicing, the treatment had a significant impact on the number (Fig. 9a, b) and volume of FL-*HTT* clusters (Supplementary Fig. 10e). PladB reduced the number of *HTT* intron1 clusters, although the effect was markedly weaker compared to that observed for FL-*HTT* (Fig. 9a, b).

As inhibition of pre-mRNA splicing leads to the nuclear accumulation of either unspliced pre-mRNA or aberrantly processed mRNA and a downregulation of fully processed mRNA, we observed a significant drop in the number of single canonically spliced FL-*HTT* molecules and a substantial increase in the number of alternatively processed *HTT* intron1 transcripts, however no effect was detected for *HTT* intron3 (Fig. 9c). Cytoplasmic FL-*HTT* mRNA puncta were downregulated, although this trend did not reach significance; conversely the levels of *HTT1a* transcripts were markedly increased (Supplementary Fig. 10f). Together these findings imply a shift in *HTT* pre-mRNA splicing toward aberrant processing under splicing-inhibitory conditions. Moreover, elevated levels of *HTT1a* in the extranuclear space are indicative of its escape from nuclear retention and its accumulation in the cytoplasmic compartment.

## Discussion

Here, we demonstrated that *HTT* nuclear RNA clusters, previously detected in YAC128 and BACHD-97Q-△N17 mice at 1 month of age^8,9^, emerge prenatally as early as embryonic day 14.5. Interestingly, FL-*HTT* clusters appeared much larger in postnatal vs embryonic animals, which was accentuated by the absence of FL-*HTT* clusters exceeding 20 μm^3^ in E14.5 animals (Fig. 2a). As development progressed, the volume of the FL-*HTT* clusters rose significantly between postnatal age P1 and juvenile P15 animals before declining in 12-month-old mice.

These results might reflect differences in the transcription rate or RNA turnover; they might also stem from a divergent RNA-binding protein repertoire or differential RNA modifications that govern RNA retention in embryo versus postnatal mice. How these various processes are regulated during neuronal development remains to be elucidated. Interestingly, the transcriptome of human embryonic stem cells (hESC) is structurally more accessible and homogeneous than that of differentiated neurons^29,30^. This greater structural flexibility could reduce the propensity of transcripts to self-associate, resulting in smaller clusters in embryonic mice. Indeed, in response to alternations in the surrounding environment (e.g. concentration of ions, metabolites, pH and osmolarity) RNA assumes various structural states (ensembles)^31,32^. Some of these states are thermodynamically favourable and can therefore become stabilised. These structural ensembles can interact in cis and/or trans with other RNA molecules either specifically or promiscuously; different RNA conformers can also scaffold various proteins^31^. In this way, RNA ensembles can interact with each other to drive the formation of bigger *HTT* nuclear RNA clusters. By contrast, *HTT* intron1 clusters were smaller and absent from the largest size bin (20–40 µm³). This is likely due to the smaller size of *HTT1a*, which may adopt a more compact conformation compared to FL-*HTT*.

Our results revealed that *HTT* nuclear RNA clusters in the brains of YAC128 mice at two months of age were enriched in the splicing proteins PRPF8 and SNARP40. Currently, it is unclear whether these clusters exacerbate RNA-mediated toxicity or if they are protective by detaining noxious transcripts in the nucleus and thus preventing their translation. Interestingly, PRPF8 and SNRNP40 are components of the U5 snRNP, a core element of the major spliceosome. The interaction of huntingtin mRNA with proteins at the core of the splicing machinery may therefore reflect active splicing and suggests a potential regulatory role for these factors in *HTT* transcript processing. Supporting this idea, a genetic screen conducted in *Caenorhabditis elegans* identified two PRPF8 alleles that strongly influence cryptic splicing by stabilising the spliceosome catalytic core^33^. Similarly, SNRNP40 deficiency has been shown to increase splicing errors, particularly intron retention, in primary hematopoietic stem cells and T cells^34^. Consistent with a broader link between *HTT* and splicing-related nuclear compartments, the phosphorylated HTT protein has previously been reported to colocalise with the nuclear speckle marker SC35^35,36^. Here, we further show that *HTT* RNA associates with MALAT1, a key architectural RNA of nuclear speckles. This previously unrecognised interaction suggests that *HTT* transcripts may be connected to speckle-associated splicing activity and may influence networks of alternative splicing events. As the mechanism and/or participating factor(s) that promote the production of *HTT1*a have not yet been elucidated, it is tempting to speculate that PRPF8/SNRNP40 could play a central role in this process. However, the formation of *HTT* nuclear RNA clusters may also have deleterious effects on nuclear homoeostasis by sequestering these and presumably other factors. Indeed, PRPF8 has already been linked to polyglutamine-mediated toxicity^37^, and in the recent report, it emerged as one of 36 splicing machinery proteins that can bind to in vitro transcribed *HTT* exon1 RNA in a CAG repeat length-dependent manner.

Liquid–liquid phase separation (LLPS) occurs when multivalent molecular interactions drive the condensation of components into distinct liquid phases with differing component concentrations. This process underlies the formation of many cellular substructures and membraneless condensates, such as paraspeckles^38^. Increasing evidence also implicates aberrant phase separation in neurodegeneration, where disease-associated proteins form dynamic condensates that can progressively transition into irreversible aggregates^38^. Although similar findings in HD remain limited^39^, it is appreciated that RNA with pathologically expanded repeat can also template multivalent intermolecular interactions. These interactions could lead to the formation of large clusters via LLPS. Indeed, Jain and Vale showed that exogenously expressed 47xCAG or 120xCAG RNA assemble into liquid-like foci, both in vitro and in the cell nucleus^14^. In contrast, our data revealed that *HTT* nuclear RNA clusters are not a result of phase separation, as treatment with 1,6-hexanediol or 2,5-hexanediol, regents that disrupt phase- separated condensates by impeding weak hydrophobic interactions, did not dissolve these structures^40^. While formation of *HTT* nuclear RNA clusters might not be driven by LLPS, examining varying concentrations and time durations of 1,6-hexanediol, as well as testing different agents that interfere with phase separation, will provide more definitive evidence.

RNA can fold into multiple secondary conformations such as hairpins, internal loops, bulges, multi-branch loops and pseudoknots^11^. Moreover, mRNAs with pathologically expanded CAG repeats form a heterogenous population of molecules with a mixture of secondary structures that are unstable and prone to self-associate or nucleate base-pairing with another RNA molecule^41^. Additionally, RNA can fold into tertiary structures, which are exceptionally sensitive to the concentrations and types of cations present in the surrounding environment^42^. The fact that *HTT* nuclear RNA clusters were disrupted by ammonium acetate underscores the involvement of monovalent cations and ionic interactions in the maintenance of clusters’ integrity and suggests that they might be composed of higher-order RNA assemblies stabilised by secondary and tertiary structures. As ionic bonding also mediates protein-RNA interactions, *HTT* nuclear RNA clusters might be further stabilised by interactions with yet unidentified nuclear protein(s), although the role of PRPF8/SNRNP40 cannot be excluded.

Building on our hypothesis that *HTT* nuclear RNA clusters could be composed of multiple interacting huntingtin transcripts, we tested doxorubicin, which disrupts intermolecular base-pairing without degrading RNA molecules. Doxorubicin markedly reduced both the number and volume of *HTT* RNA clusters and produced a biphasic effect on nuclear and cytoplasmic FL-*HTT* mRNA levels, with an initial increase followed by a drop after 10 h. This initial increase might reflect greater availability of transcripts after cluster disassembly and their nuclear egress, whereas the later decrease likely results from doxorubicin-mediated interference with transcriptional machinery. In contrast, nuclear and cytoplasmic *HTT1a* mRNAs showed a sustained, time-sensitive downregulation, consistent with a stronger disruption of *HTT1a* clusters than FL*-HTT* clusters. The mechanism underlying the distinct effects of doxorubicin on FL-*HTT* and *HTT1a* remains unclear. Given that doxorubicin can alter the localisation and function of RBPs involved in splicing and polyadenylation^43,44^, generation of *HTT1a*, which depends on noncanonical processing events, may be particularly vulnerable to such perturbations.

Doxorubicin, however, has pleiotropic effects: its intercalation into the DNA double helix causes blockage of transcriptional elongation and inhibition of topoisomerase II, impacting chromatin structure^45,46^. Therefore, future studies are warranted to determine which of these doxorubicin-mediated events lead to the disassembly of *HTT* nuclear RNA clusters.

Transcriptional blockage led to a marked decrease in the number of *HTT* nuclear RNA clusters, an effect observed with three different transcription inhibitors, two of which (DRB, NVP-2) specifically hinder the elongation phase. These findings suggest that unperturbed de novo synthesis of huntingtin mRNA is indispensable for their effective assembly into nuclear clusters and further indicate that these structures are dynamic rather than solid-like. Notably, Pol II transcription processivity is regulated by C-terminal domain phosphorylation, which modifies condensate partitioning and redistribution of Pol II between initiation-, elongation- and splicing-associated condensates^38,47^. Although our findings suggest that phase separation is not the primary driver of *HTT* nuclear RNA clusters assembly, inhibition of transcriptional elongation may still promote their dispersion by disrupting elongation-associated condensates.

Transcriptional inhibition drives relocalisation of numerous RBPs, including TDP-43, from the nucleus to the cytoplasm^28^. If *HTT* RNA clusters interact with proteins in the nucleoplasm, nuclear efflux of these factors might affect *HTT* nuclear RNA clusters’ stability. Importantly, in our study, TDP-43 was not identified as an interactor of *HTT* nuclear RNA clusters (Supplementary Fig. 6), however other proteins such as HuR, hnRNPA1, NFX1 also translocate to the cytoplasm in response to transcriptional inhibition^28^. Mapping the proteome of *HTT* nuclear RNA clusters will clarify the role of RBPs in maintaining their integrity.

Strikingly, transcriptional inhibition led to intranuclear accumulation of single FL-*HTT* and *HTT1a* mRNAs, which increased the number of transcripts amenable to nuclear transport. These single mRNAs likely arise from the disintegration of *HTT* nuclear RNA clusters, as transcriptional block would otherwise decrease overall mRNA levels. Importantly, elevated cytoplasmic transcripts could then be translated into toxic proteins, potentially exacerbating cellular dysfunction.

We show that *HTT* nuclear RNA clusters can be detected in YAC128 embryos and that they persist into late adulthood (up to 12 months), although their composition may change over time. These structures are predominantly observed in neurons rather than glia and are dynamic, as their integrity is disrupted by the inhibition of ionic interactions, transcription, or splicing. The functional significance of these clusters remains unclear. Nuclear retention of mutant *HTT* mRNA could potentially limit the production of toxic protein isoforms; however, expanded CAG-repeat RNA itself has been implicated in polyQ pathogenesis through the formation of hairpin structures that sequester key RNA-binding proteins, such as MBNL1, leading to splicing dysregulation^48,49^, as observed in myotonic dystrophy^48,50^. Whether *HTT* RNA clusters interact with MBNL1 remains unresolved, as available antibodies did not yield a detectable signal. Notably, published data suggest that *HTT* nuclear clusters are resistant to ASO treatment, highlighting the need to define their function and determine whether they occur in other HD models, including human tissue, to inform the development of RNA-targeted therapeutics.

## Methods

### Ethics

All procedures were performed in accordance with the Animals (Scientific Procedures) Act, 1986, and approved by the University College London Ethical Review Process Committee. We have complied with all relevant ethical regulations for animal use.

### Animal colony breeding and maintenance

YAC128 mice were maintained on a C57BL6/J (Charles River, UK) background. Mice were group-housed depending on gender, and genotypes were mixed within cages. All animals were kept in individually ventilated cages containing Aspen Chips 4 Premium bedding (Datesand) with environmental enrichment in the form of chew sticks and a play tunnel (Datesand). All mice had ad libitum access to water and chow (Teklad global 18% protein diet, Envigo, The Netherlands). The temperature was automatically regulated at 21 °C ± 1 °C, and animals were kept on a 12 h light/dark cycle. The animal facility was barrier-maintained, and quarterly non-sacrificial FELASA (Federation of European Laboratory Animal Science Associations) screens found no evidence of pathogens. Equal numbers were collected for males and females at all ages. Animals were sacrificed at postnatal day 1 and 15, as well as 2 and 12 months of age. Female mice carrying embryos at approximately 14.5 days were sacrificed at 5 months. The uterine horn was dissected and separated into individual embryos, and the embryos were released from the embryonic sac. Thereafter, the head of the embryo was cut to separate it from the body; a tail cut was taken into a separate Eppendorf for genotyping. Two templar incisions were made by inserting the tips of the scissors as to not cut through the brain; then, watchmakers were used to gently lift the skull and separate it from the brain. Once the brain was exposed, it was lifted from below to remove it from the head. This process was repeated for all YAC128 animals sacrificed; however, from P15 onwards the brains were separated into hemispheres, which were then embedded individually in Optimal Cutting Temperature (OCT) compound (Cell Path, KMA-0100-00A). Mouse embryos and postnatal day 1 pups were euthanized by decapitation, whereas older mice were euthanized by cervical dislocation.

### DNA extractions, genotyping and repeat sizing

Genomic DNA from embryonic day 14.5 animals and postnatal day 1 pups was extracted from the tail cut. Genomic DNA from postnatal day 15 pups and 2, 12-month-old mice was obtained from ear notches, and genotyping was performed as previously described^51^. Briefly, PCR reactions were set up (5 μl DreamTaq MasterMix (Thermofisher Scientific, K9022), 1 μl of primer 1, 2, 3 and 4 (10 μM)) (Table 1) into 96-well plate, and 1 μl of DNA template (50 ng/μl) per well was added. Control templates were included (positive (YAC128 TG), YAC128 negative (WT), a H_2_O well and a blank no-template well). PCR Programme was run (3 min at 95 °C, 34 cycles of 30 s at 95 °C, 30 s at 60 °C and 1 min at 72 °C, and final elongation step cycle for 15 min 72 °C) and the PCR reaction was visualised using gel electrophoresis (2% agarose gel, ran for 30 min at 130 V), in 1× TAE (Tris-acetate-EDTA) solution.

**Table 1:** Primer sequences used for genotyping

Primer name	Primer sequence from 5′ to 3′	Amplicon size (transgenic positive)	Amplicon size (WT positive)
Primer 1	AGCCCTACACTAGTGTGTGTTACACA	450 base pairs	450 base pairs
Primer 2	CTTGTTGAGAACAAACTCCTGCAGCT		
Primer 3	CGCAGGCTAGGGCTGTCAATCATGCT	273 base pairs	No band
Primer 4	TCATCAGCTTTTCCAGGGTCGCCAT		

The polyQ repeat of 125 glutamines in YAC128 mice is encoded by (CAG)_23_(CAA)_3_CAGCAA(CAG)_80_(CAA)_3_CAGCAA(CAG)_10_CAACAG, which is stable on germline transmission^52^.

### Primary neuronal cultures

For the preparation of primary hippocampal cultured cells derived from neonatal YAC128 mice and their wild-type siblings, postnatal animals (day P0–P2) were sacrificed by decapitation. Subsequently, hippocampi were isolated, deprived of meninges and collected into 2 mL Eppendorf containing dissection medium (HBSS (Gibco, 14175-053) supplemented with 1× sodium pyruvate (Gibco, 11360-070), 20% glucose (Sigma, G8270-1KG),1 M HEPES pH = 7.3 (Gibco, 15630-080), thereafter hippocampi were washed twice with dissection medium and 1 mL of papain solution containing 60 µL of DNase was added (per two hippocampi; papain dissociation system kit, Worthington Chemical Corporation, LK003150). After 15 min incubation at 37 °C, the tissue was washed once with warm dissection medium and twice with warm plating medium (DMEM (Gibco, 41966-029) supplemented with 10% FBS (Gibco, 16140-071), 20% glucose, 1× sodium pyruvate, 1× glutamax (Gibco, 35050-061) and penicillin/streptomycin (Gibco, 15070-063)) and then subjected to mechanical tituration first with 1 mL pipette and then with 100 µL pipette. In the next step, tissue was centrifuged at 100 rcf for 5 min at RT, the supernatant was discarded, and cells resuspended in 670 µL EBBS with 75 µL albumin-ovomucoid inhibitor and 35 µL DNase (per animal, papain dissociation system kit). The mixture was layered atop 1.25 mL of albumin-ovomucoid inhibitor and centrifuged at 1880 rcf for 6 min at RT. The cell pellet was resuspended in 500 µL of plating medium, and equal volumes of the resuspended cells and Trypan Blue solution were mixed. Cell viability and number were then determined using the Tecan cell counting method. Thereafter, cells were diluted into plating medium and plated onto poly-D-lysine-coated (R&D Systems, 3439-100-01) glass coverslips (VWR, 631-0149), placed into a 24-well plate (Falcon, 353947). After 2 h, 1 mL of maintenance medium (Neurobasal medium (Gibco, 21103-049) supplemented with B27 (Gibco, A35828-01), 1× glutamax and penicillin/streptomycin) was added to each well of a 24-well plate and placed in a humidified incubator. Cells were plated at a density of 150,000/coverslip and cultured for 18–10 days in vitro (DIV) unless stated otherwise.

### RNAscope analysis and quantification

Whole brains (embryonic day 14.5; postnatal day 1 and 15) or brain hemispheres (2, 12 months) from YAC128 mice were harvested, embedded in optimal cutting temperature compound (OCT, CellPath Ltd) and stored at −80 °C until further use. Brain blocks were equilibrated to about −30 °C in a cryostat (Bright Instruments), cut into 20 µm thick sagittal sections and mounted onto SuperfrostPlus slides (VWR, 25 × 75 × 1.0 mm). The brain sections, or cells cultured on coverslips, were fixed in 4% paraformaldehyde (Pioneer Research Chemical Ltd) for 20–30 min at 4 °C, followed by dehydration in an increasing concentration of ethanol, starting at 50%, moving to 70% and finishing with 100%, each time for 5 min (for brain sections) or 1 min (for cultured cells) at RT. Next, a hydrophobic barrier was created around each section with a hydrophobic barrier pen (ImmEdge Pen, Vector; H-4000) and allowed to air-dry for about 1 min. About five drops of 3% hydrogen peroxide solution (H_2_O_2_) was added onto the section and incubated for 10 min at RT, followed by three washes with PBS. Protease IV (for brain sections) or protease III (for cultured cells, diluted 1:20) was then added to the samples and incubated for 30 min (for brain sections of animals at 2 and 12-months of age), 15 min (for brain sections of animals at embryonic day 14.5; postnatal days 1 and 15) or 10 min (for cultured cells) at RT. The protease was removed, and the slides submerged in PBS three times for 5 min each.

The hybridisation and amplification steps were performed in accordance with the RNAscope protocol (Advanced Cell Diagnostics, Inc.). Briefly, probes were hybridised for 2 h at 40 °C in a HybEZ oven (Advanced Cell Diagnostics, Inc., 220 VAC, 310013). RNAscope probes used included: Mm-Malat1 (ACD, 313391), Mm-Neat1 (ACD, 440351), full-length human *HTT* exons 14-61 (ACD, 473201); intron 1 human *HTT* (ACD, 561431), intron 3 human *HTT* (ACD, 1278811), intron 45 human *HTT* (ACD, 1278821). Following each amplification step, the slides were washed twice with PBS for 5 min each at RT. Each probe was developed in three steps: the HRP (horse radish peroxidase) step, the TSA (tyramide signal amplification) fluorophore step and the HRP blocker step, using the RNAscope Multiplex Fluorescent v2 Assay. Each step was followed by two washes in PBS for 5 min at RT. A different TSA fluorophore (PerkinElmer) was assigned to each probe channel. The following fluorophores were used at 1:1500 dilution: fluorescein, Cy3 and Cy5. Subsequently, the slides were counterstained with DAPI for about 5 min at RT. Tissue section slides were mounted in VECTASHIELD Vibrance Antifade Mounting Media (Vector Laboratories, H-1000-10), a 24 × 60 mm glass coverslip (VWR International, ECN 631-1575) was placed over the tissue section, and the slides were air dried before being imaged. Cells on the coverslips were mounted in EverBrite ^TM^ hardset mounting medium (Biotium, 23003) and air-dried before imaging. Images were acquired with an inverted (Zeiss LSM980) confocal integrated microscope system with a 63× objective (HC PL APO 63×/1.40 Oil).

### Immunofluorescence

Immunofluorescence was conducted in conjunction with the RNAscope protocol. For combined RNAscope-IF experiments, primary antibodies were diluted 1:10 in Co-Detection Diluent (Advanced Cell Diagnostics, 323160) and were applied after the hydrogen peroxidase step and incubated overnight at 4 °C. Primary antibodies included: NeuN (Abcam, ab177487), GFAP (Invitrogen, PA1-10004), PrPF8 (Abcam, ab79237), coilin (Abcam, ab11822), SFPQ (Abcam, ab38148), H3K9ac (Upstate, cell signalling solutions06-942), p84 (GeneTex, GTX118740), TDP-43 (Abcam, ab104223), hnRNP K (Abcam, ab52600), hnRNP F (Thermofisher Scientific, CF500804), SNRNP40 (Atlas Antibodies, HPA038904). The following day, cells were washed twice with PBS and subjected to the regular RNAscope procedure described above. Before DAPI staining, secondary antibodies were applied at RT for 2 h.

Secondary antibodies were diluted in Co-Detection Diluent, used at 1:200 and included anti-mouse (Alexa555, Invitrogen, A32773), ani-rabbit (Alexa555, Invitrogen, A32794), anti-goat (Alexa488, Invitrogen, A11055).

### Hippocampal cell treatments

Five percent or 8% 1,6-hexanediol (Sigma, 240117-50 G) and 8% 2,5-hexanediol (Merck, 240117-50 MG) or an equivalent dilution of water as control were added to hippocampal cells in culture for 5 min. Ammonium acetate (Sigma, A1542-250 G) was added to the cultured media and cells were incubated in this medium for 10 min. Cells were treated with transcriptional inhibitors: Actinomycin D (Sigma, A1410-2 MG), NVP2 (Tocris, 6535) and DRB (Millipore, 287891-50 mg) for 3 h. Splicing blocker, PladB (Cayman Chemical Company, 364-9897) was added to hippocampal cells in culture for 4 h. Cells were incubated with doxorubicin (Cell signalling Technology, 5927S) for either 5 or 10 h. Unless otherwise stated DMSO (Sigma, D2438-10 ML) served as a control. YAC128-derived hippocampal cells and their control counterparts were used at 18–20 DIV.

### Analysis of RNA clusters/ transcripts

We used a size-based threshold to identify RNA clusters/puncta. Briefly, YAC128 sections were imaged using a confocal microscope, and 1 μm Z-stacks were acquired. For YAC128 hippocampal cells, Z-stacks at 0.5 μm were acquired. Images were converted to 8-bit, and a DAPI signal was used to create a threshold-based mask for cell nuclei to separate nuclear and cytoplasmic signals for different huntingtin transcripts. To create a Z-stack of molecules outside of the nucleus, an 8-bit Z-stack containing images of one of the three *HTT* transcripts was subtracted from the binary DAPI mask Z-stack. To generate a Z-stack of molecules inside of the nucleus an 8-bit Z-stack containing images of one of the three *HTT* transcripts was subtracted from the previously created out-of-the-nucleus Z-stack. RNA clusters and nuclear puncta, as well as cytoplasmic mRNA puncta, were identified using the FIJI 3D Objects Counter plugin^53^. An intensity threshold in this plug-in was set to segment objects from the background, with the intensity threshold varying between different treatments but kept constant for control and treatment within the same experimental group. Thereafter, the size filter was set to the minimum of about 10 voxels to filter out noise, and the maximum was set automatically by the programme to include large nuclear structures. This plug-in faithfully identified clusters/single mRNAs as depicted in Supplementary Fig. 2a. The method was applied to determine the number, volume and surface area of the clusters/ single mRNAs. The number as well as the total volume taken up by the clusters per cell were chosen as the parameters to quantify the extent of cluster formation.

### Colocalisation analysis

Manders’ coefficients as well as colocalisation coefficient 1 (corresponding to the *HTT*/ *HTT1a* cluster channel) and colocalisation coefficient 2 (corresponding to the nuclear marker channel), presented in Fig. 4b, were calculated from Z-stack images using ZEISS ZEN Blue 3.9 (ZEISS) following Costes automatic thresholding to minimise the contribution of background signal. To further assess the spatial relationship between *HTT* nuclear clusters and nuclear markers, the colocalisation colormap plugin in Fiji was used. This method evaluates colocalisation locally at the pixel level after intensity weighting and thresholding and generates a spatial map of correlation values across the image, allowing visualisation and quantification of regions where both signals are simultaneously present. The analysis produces an index of correlation (IC) derived from the distribution of normalised mean deviation product (nMDP) values. For the analysis shown in Fig. 4a, *HTT* nuclear clusters were first segmented in ImageJ using the analyze particles function, following intensity thresholding to identify cluster objects and exclude single *HTT* mRNA molecules. The resulting binary masks defining *HTT* cluster regions were then used for subsequent colocalisation colormap analysis to calculate the index of correlation.

### Statistical analysis

The results are expressed as the mean ± SEM. To test the data for normal distribution Shapiro–Wilk test was applied. Whenever normality requirements were not met, a non-parametric test was used. For experiments determining developmental trajectory of clusters appearance either one-way ANOVA followed by a post-hoc Tukey’s test (parametric) or Kruskal–Wallis with Dunn’s correction for multiple comparisons (non-parametric) were used. For experiments on hippocampal cells comparing the effect of different pharmacological agents on the clusters/transcripts number/volume, statistical significance was analysed using either unpaired, two-tailed t-test or unpaired, two-tailed Mann–Whitney test. Statistical analyses were performed using GraphPad Prism 10 (GraphPad Software Inc.). The statistical tests used and sample size for each analysis are shown in the Figure legends.

### Reporting summary

Further information on research design is available in the Nature Portfolio Reporting Summary linked to this article.

## Supplementary information

Transparent Peer Review File

Supplementary Information

Description of Additional Supplementary Files

Supplementary Data 1

Supplementary Data 2

Reporting Summary

## Data Availability

All data that support the findings of this study are included in this publication and its Supplementary Information files. The source data underlying the figures can be found in Supplementary Data 1 and 2.
