# Supplementary material for: Nuclear RNA clusters are dynamic structural entities in Huntington’s disease
Source: Commun Biol. 2026 Jun 29;9:1370. doi: 10.1038/s42003-026-10419-1 (PMC13680494; doi:10.1038/s42003-026-10419-1)
Supplement: Supplementary file 1 [file 42003_2026_10419_MOESM1_ESM.pdf]

nature portfolio

Peer Review File

Nuclear RNA clusters are dynamic structural entities in Huntington's Disease.

Corresponding Author: Dr Sandra Fienko

This file contains all reviewer reports in order by version, followed by all author rebuttals in order by version.

Version 0:

Reviewer comments:

Reviewer #1

(Remarks to the Author)
This is an interesting manuscript that addresses a timely topic of the role of mutant HTT RNA in HD pathogenesis in vivo which is especially important since HTT RNA has emerged as a major target in current preclinical studies and clinical trials. The authors demonstrate the presence of HTT RNA clusters in the prenatal stage at day 14.5, indicating early developmental effects in HD YAC128 model. They applied various pharmacological treatments in YAC128-derived hippocampal cultures and demonstrate that the assembly of HTT RNA clusters is influenced by ionic interactions and that these clusters can be dissolved upon application of nucleic acid intercalators. They show that unperturbed transcription nucleates these structures and that blockage of canonical splicing disrupts HTT nuclear RNA clusters. These data present compelling evidence of the dynamic nature and malleability of HTT nuclear RNA clusters which may influence their accessibility to pharmacological and therapeutic interventions. The experiments are well-designed and carefully executed providing important novel characterization of HTT RNA clusters in HD mouse model. The quantification and statistical analysis is adequate for most data sets.
Despite these strengths, the study may have limited impact as it falls short of highlighting a novel mechanism of HTT RNA toxicity or suggesting specific recommendations towards effective ASO design. In addition, some of the conclusions, ("these clusters were confined to neurons, implying a neuron-specific mechanism of accumulation, and were colocalized with Prpf8, a core spliceosomal protein"), are not well supported by the data, especially immunofluorescence data is problematic (see below).
--- Co-localization analysis with PRPF8 and nuclear bodies (Figures 4 and supplemental Figure 6) is not convincing.. It is not supported by adequate quantitation of co-localization.
The authors use colormap plug-in in ImageJ software19 and conclude that PRPF8 interacted strongly with HTT intron 1 (Icor=0.65) and FL-HTT (Icor=0.64). However, no colocalization has been suggested with several nuclear bodies based on Icor ranging from 0.37 to 0.54 (Suppl Fig 6). Visually it is hard to judge since most images on Fig. 4 and S6 are oversaturated and there is no PRPF8 single label shown.
The authors suggest association of RNA clusters with splicing machinery based on co-localization with a single splicing factor PRPF8. Also lacking is co-localization analysis with major splicing-related organelle, nuclear speckles (NS). Association of HTT protein with NS have been shown previously and it would be interesting to assess HTT RNA clusters in this regards.
Thus, one of main conclusions of the study, that Huntingtin RNA clusters associate with RNA splicing machinery but do not accumulate nearby well-characterized nuclear bodies, is not well supported by the data and needs more careful co-localization quantitation analysis (e.g. with ZEN software including Pearson's and Mandel coefficients), more conclusive images and preferably additional data with NS and other splicing factors
---The authors conclude that RNA clusters were confined to neurons, implying a neuron-specific mechanism of accumulation. "The FL-HTT probe revealed large RNA clusters detained in the cells of neuronal origin but not in the glial cells which mainly contained single FL-HTT mRNAs (Supplementary Fig. 2b)."
This conclusion is not supported by the data shown on Supplementary Fig 2b. It is not possible to distinguish large clusters from single mRNAs from the images shown. Images at higher magnification and some kind of quantitation should be included to support this.
The same issue in Figure 4C- the data need to be quantitated to draw the conclusion
---Images on Fig 6a are not representative for HTT intron 1 and 3 as similar few RNA clusters are visible in treated and control cells, whereas quantitative data shows a drastic decrease with 10h treatment
---The inset position is not clearly visible in most figures

--- The following statement in the abstract -"Huntington's disease research has focused on a toxic protein gain-of-function as the main driver of pathology"-does not consider the loss of normal function of HTT as potential mechanism, suggested by several studies. This should be noted.
---The "Conclusion" section does not really summarize conclusions directly arising from the data shown, but rather present hypotheses of HTT RNA toxicity based on previous studies. These thoughts would be more appropriate within general Discussion or Introduction, unless the authors can directly connect their data with proposed conclusions.

Reviewer #2

(Remarks to the Author)
This manuscript characterizes HTT RNA nuclear clusters in the brains of pre- and post-natal YAC128 mouse brains, and in primary mixed cultures. The authors show the clusters are present in all tested model systems, and at all tested developmental time points. The manuscript claims the clusters may contribute to HD pathophysiology by disrupting RNA splicing in neurons, and may inform targeting strategies used by future HD therapeutics. The manuscript demonstrates that HTT RNA nuclear clusters are detectable in prenatal HD mouse model brains, which is a novel finding that contributes to the field's understanding of HD pathogenesis. In particular, the developmental aspect of HD biology is relatively understudied, and this manuscript will make a valuable contribution to this area of HD research. Association of the clusters with splicing factor PRPF8 also adds to existing work from this group that explores aberrant splicing of HTT mRNA as a contributor to HD pathophysiology. The impact of CAG-repeat containing mRNA on nuclear dynamics may also be of interest to wider audiences focused on other CAG repeat disorders, as the authors mention in the text. In general, the manuscript is logically organized, the experiments are explained well, and descriptions of the statistics are thorough and the tests used are appropriate. The conclusions of the work are original (although a bit preliminary). This work elucidates a novel aspect of HD pathophysiology that contributes to the growing body of evidence suggesting RNA toxicity is important in HD. It is likely this work will support further investigation of RNA-associated disease mechanisms in the HD field.

1. The manuscript would benefit from general editing to improve readability and address minor grammar and punctuation errors.

2. The authors present a number of hypotheses in the discussion exploring mechanistic drivers of RNA cluster formation and stability; however, these are not followed up or explored with experiments in the manuscript, and doing so would increase the novelty and impact of the work. For example, m6A modification could be measured and correlated with RNA cluster dynamics in pre- and post-natal mice. The impact of destabilizing the clusters upon an HD-associated cellular phenotype could also be assessed to explore the putative contribution of HTT RNA clusters to HD pathogenesis.

3. The introduction references the presence of nuclear HTT RNA clusters in the brains of YAC128 and BACHD mice, and states that this evidence indicates the RNA clusters only form when human huntingtin transcripts are present. Has a study been done that shows these clusters aren't present in a full-length murine KI HD model (e.g., the Hdh(CAG)150 model)? If so, it should be referenced here. If not, this study should be conducted, or the claims in this section should be tempered to accurately reflect existing data.

4. The volume ranges used to distinguish between single nuclear mRNA transcripts and RNA clusters overlap. Please modify the bin boundaries to correct this.

5. It would be helpful to fully label the y-axes in Fig. 1c-e, as the lower bounds of the second and third y-axis segments are not numbered. It also appears that the measurements reported in the text don't match the plotted measurements in this figure (e.g., early postnatal and adult mice appear to have FL-HTT clusters that measure <40 um^3 based on the plotted data, but the text reports these measurements to reach up to 45 um^3). Similarly, the largest clusters measured in E14.5 mice (in Fig. 1c) all appear to be <10 um^3. The data distribution is also different in the corresponding supplementary figure, but it is unclear what the difference is between the two figures (e.g., it is reported that the primary figure shows averages, but how were these data computed?). There are also >3 data points for each animal group, but N=3 is reported in the figure caption.

6. On page 4, the authors state size changes in RNA clusters over time suggest "a dynamic regulation of cluster size across developmental and aging stages". This statement suggests this is an active process/mechanism that regulates cluster size in YAC128 brain over time. Is this accurate, or could cluster size fluctuations be a consequence of other processes?

7. In Fig. 4, the inset on the overview that indicates the sampled area in remaining panels is very difficult to see – could this be changed so the inset is easier to distinguish?

8. On page 8, a concluding sentence reads "these results suggest that HTT nuclear RNA cluster assembly is partly regulated by ionic interactions mediated by proteins that neutralize negatively charged RNA molecules". The first part of this statement is supported by the data, but there is not biological evidence to suggest proteins that interact with negatively-charged RNA mediate the formation of RNA nuclear clusters, as chemical treatments were used to manipulate cluster morphology. Please temper this claim or include an additional experiment that specifically addresses the biological mechanism(s) driving this observation.

9. The last sentence of the Results states that "elevated levels of HTT1a in the extranuclear space are indicative of its escape from nuclear retention and its accumulation in the cytoplasmic compartment". However, the figures referenced in the previous sentences (Fig. 9c and Supp. Fig. 9f) show increases in HTT1a transcripts both inside and outside the nucleus – is

this possible?

Version 1:

Reviewer comments:

Reviewer #1

(Remarks to the Author)
This revised manuscript now provides several new controls and additional images that help to increase the rigor of the study. The authors have included additional data supporting the conclusion that nuclear clusters are associated with nuclear RNA-processing machinery. The conclusion section has been revised to better summarize the main findings. The manuscript still lacks mechanistic studies towards understanding HTT RNA toxicity, although this perhaps does not preclude the publication.

Reviewer #2

(Remarks to the Author)
The authors have carefully and thoroughly revised this manuscript in response to all reviewer comments. Additional explanations of the analytical methods have significantly improved readability of the manuscript for non-specialist audiences (including this reviewer, who is not an expert in RNA biology) and changes to the figures have made them easier to interpret. The revised manuscript will make a valuable contribution to the HD field and may inform future studies of HD therapeutics designed to target HTT mRNA.

Open Access This Peer Review File is licensed under a Creative Commons Attribution 4.0 International License, which permits use, sharing, adaptation, distribution and reproduction in any medium or format, as long as you give appropriate credit to the original author(s) and the source, provide a link to the Creative Commons license, and indicate if changes were made.
In cases where reviewers are anonymous, credit should be given to 'Anonymous Referee' and the source.
The images or other third party material in this Peer Review File are included in the article's Creative Commons license, unless indicated otherwise in a credit line to the material. If material is not included in the article's Creative Commons license and your intended use is not permitted by statutory regulation or exceeds the permitted use, you will need to obtain permission directly from the copyright holder.
To view a copy of this license, visit https://creativecommons.org/licenses/by/4.0/

Reviewer #1 (Remarks to the Author):

This is an interesting manuscript that addresses a timely topic of the role of mutant HTT RNA in HD pathogenesis in vivo which is especially important since HTT RNA has emerged as a major target in current preclinical studies and clinical trials. The authors demonstrate the presence of HTT RNA clusters in the prenatal stage at day 14.5, indicating early developmental effects in HD YAC128 model. They applied various pharmacological treatments in YAC128-derived hippocampal cultures and demonstrate that the assembly of HTT RNA clusters is influenced by ionic interactions and that these clusters can be dissolved upon application of nucleic acid intercalators. They show that unperturbed transcription nucleates these structures and that blockage of canonical splicing disrupts HTT nuclear RNA clusters. These data present compelling evidence of the dynamic nature and malleability of HTT nuclear RNA clusters which may influence their accessibility to pharmacological and therapeutic interventions. The experiments are well-designed and carefully executed providing important novel characterization of HTT RNA clusters in HD mouse model. The quantification and statistical analysis is adequate for most data sets. Despite these strengths, the study may have limited impact as it falls short of highlighting a novel mechanism of HTT RNA toxicity or suggesting specific recommendations towards effective ASO design. In addition, some of the conclusions, ("these clusters were confined to neurons, implying a neuron-specific mechanism of accumulation, and were colocalized with Prpf8, a core spliceosomal protein"), are not well supported by the data, especially immunofluorescence data is problematic (see below).

1. Co-localization analysis with PRPF8 and nuclear bodies (Figures 4 and supplemental Figure 6) is not convincing. It is not supported by adequate quantitation of co-localization. The authors use colormap plug-in in ImageJ software19 and conclude that PRPF8 interacted strongly with HTT intron 1 (Icor=0.65) and FL-HTT (Icor=0.64). However, no colocalization has been suggested with several nuclear bodies based on Icor ranging from 0.37 to 0.54 (Suppl Fig 6).

We thank the reviewer for this comment. The Icor values reported in Supplementary Fig. 6 range from 0.37 to 0.54. Values in this range are generally interpreted as indicating **weak to moderate spatial association rather than robust colocalisation**. The value of 0.37 is relatively low and consistent with the absence of meaningful overlap between the signals. Although the value of 0.54 is somewhat higher, it still falls within the range typically considered moderate and does not indicate strong colocalisation. In fluorescence imaging analyses, true colocalisation is usually reflected by higher correlation values.

2. Visually it is hard to judge since most images on Fig. 4 and S6 are oversaturated and there is no PRPF8 single label shown.

We have now included images where single PRPF8 label is shown (in addition to single label SNRNP40, Malat1 and Neat1) in the new Supplementary Figure 6.

3. The authors suggest association of RNA clusters with splicing machinery based on co-localization with a single splicing factor PRPF8. Also lacking is co-localization

analysis with major splicing-related organelle, nuclear speckles (NS). Association of HTT protein with NS have been shown previously and it would be interesting to assess HTT RNA clusters in this regard. Thus, one of main conclusions of the study, that Huntingtin RNA clusters associate with RNA splicing machinery but do not accumulate nearby well-characterized nuclear bodies, is not well supported by the data and needs more careful co-localization quantitation analysis (e.g. with ZEN software including Pearson's and Mandel coefficients), more conclusive images and preferably additional data with NS and other splicing factors.

We thank the reviewer for this suggestion and agree that the Pearson correlation coefficient (PCC) is widely used in fluorescence colocalisation studies. However, PCC quantifies the linear correlation of intensities between two channels across all pixels in the image. As such, it measures whether the two signals vary together globally, but it does not directly measure spatial overlap between structures. This metric performs poorly when one signal is homogeneously distributed and the other forms sparse puncta**,** because the large number of pixels with near-zero intensity in the punctate channel and the low variance of the diffuse signal bias the coefficient toward zero, even when puncta reside within regions containing the diffuse protein. **Thus, PCC does not reliably capture this type of spatial relationship.**
The Manders' overlap coefficient partially addresses this limitation (**included in the Fig. 4b**) by quantifying the fraction of thresholded signal that overlaps between channels. However, Manders yields a single global value and does not indicate where within the image colocalisation occurs. In contrast, the colocalisation colormap approach based on the Normalized Mean Deviation Product (nMDP) evaluates colocalisation locally at the pixel level, after intensity weighting and thresholding, thereby identifying where both signals are simultaneously present and to what extent. This approach reduces the influence of background pixels and provides a **spatially resolved representation of colocalisation,** allowing the overlap to be directly related to identifiable subcellular structures. Because our biological question concerns whether the two markers occupy the same subcellular structures, rather than whether their global intensities covary, this spatially resolved analysis is more appropriate.
Furthermore, now in Fig. 4a we performed colocalisation colormap analysis after segmentation of the clusters, directly quantifying their overlap with nuclear markers of interest. Following the reviewer's suggestion, we also included additional nuclear markers, including the **nuclear speckle-associated lncRNA MALAT1** (visualised by RNAscope), the **splicing factor SNRNP40**, and paraspeckle marker NEAT1. We observed strong spatial association with MALAT1 and SNRNP40, but not with NEAT1, further supporting the conclusion that the nuclear clusters are associated with key nuclear RNA-processing machineries.
We also quantified the overlap coefficient (ColocCoefficiency1 ColocCoefficiency2) (Fig4b). In this analysis, the coefficient corresponding to the nuclear clusters (ColocCoeff1) ranged from ~0.40–0.50, indicating that **approximately half of the cluster signal overlaps with the nuclear proteins analysed**. In contrast, the reciprocal coefficient corresponding to the nuclear protein channel (ColocCoeff2) was substantially lower (~0.20 and as low as ~0.05 for other proteins). This asymmetry is

expected given the spatial organisation of the signals: the nuclear proteins are **broadly distributed throughout the nucleus**, whereas the clusters represent **discrete structures occupying only a small fraction of the nuclear area**. Consequently, while a significant proportion of cluster signal can coincide with the nuclear proteins (moderate ColocCoeff1 values), only a small fraction of the total nuclear protein signal is located within clusters (low ColocCoeff1 values). These values are therefore consistent with the expected geometry of **punctate structures embedded within a broadly distributed nuclear signal**, and further support the spatial association detected by the colocalisation colormap.

The revised text is included in the manuscript (lines: 171-224):

The nuclear interior is organized around chromatin into specific, microscopically visible nuclear substructures, called nuclear bodies which play a distinct role in RNA metabolism. To understand the molecular identity of *HTT* nuclear RNA clusters, we combined RNAscope and immunofluorescence (RNAscope-IF) and stained YAC128 brain sections at 2 months for markers of well-identified nuclear bodies. Given that these structures contain *HTT*intron 45 and are adjacent to transcription sites[9], we hypothesised they could be localised near the splicing machinery. Several proteins captured by mutant *HTT* RNA belong to the spliceosome pathway, including PRPF8 and SNRNP40 [15, 18], which also interact with *in vitro* transcribed *HTTexon1*[18]. RNAscope-IF revealed robust colocalization of PRPF8 with *FL-HTT/HTTintron1-rich clusters* (Fig. 4a, Supplementary Fig.6a). Subsequent analysis, using the Colocalisation colormap plug-in in ImageJ software, confirmed a strong spatial association of PRPF8 with *HTTintron 1* (Icor=0.65) *and FL-HTT* (Icor=0.64) nuclear clusters, comparable in strength to the binding between *HTTintron 1 and FL-HTT* (Fig. 4a, Icor=0.67). To strengthen the evidence that *HTT* nuclear clusters are associated with the splicing machinery, we examined another spliceosomal component, SNRNP40. PRPF8 and SNRNP40 are known to functionally interact, and both proteins are essential for efficient pre-mRNA splicing. Consistently, *HTT* nuclear clusters showed strong spatial association with SNRNP40 (Fig. 4b, Supplementary Fig.6b, Icor=0.62), supporting the idea that these structures are positioned within sites enriched in RNA processing factors. We next investigated whether *HTT* nuclear clusters interact with long non-coding RNAs: MALAT1, a component of nuclear speckles involved in splicing regulation, and NEAT1, the architectural RNA of paraspeckles implicated in gene regulation. *HTT* nuclear clusters displayed clear association with MALAT1 (Fig. 4b, Supplementary Fig.6b, Icor = 0.62) but not with

NEAT1 (Fig. 4b, Supplementary Fig.6c, Icor = 0.18), suggesting that *HTT* clusters preferentially localize to splicing-related nuclear domains rather than paraspeckles. To complement the spatial analysis, we quantified **Manders' Colocalization Coefficient (MCC)**, as well as Colocalisation Coefficiency1 and Colocalisation Coefficiency2 (Fig. 4c). **MCC** measures the fraction of signal that coincides between two channels[23, 24]. The MCC values obtained (~0.40–0.50) indicate a **moderate degree of overlap** between *HTT* nuclear clusters and the nuclear markers analysed. Because MCC reflects global signal overlap and does not capture spatial distribution, these values were interpreted alongside colocalisation colormap analysis, which provides spatially resolved information. The colocalization coefficiency corresponding to the *HTT* nuclear cluster channel (ColocCoefficient1) ranged from ~0.40–0.50, indicating that approximately half of the cluster signal overlaps with the analysed nuclear markers. In contrast, the reciprocal coefficiency for the nuclear protein channel (ColocCoefficient2) was substantially lower (~0.20 and as low as ~0.05 for some proteins). This asymmetry indicates that while a considerable fraction of the cluster signal coincides with the nuclear markers, only a small proportion of the total nuclear protein signal resides within clusters. These values are consistent with punctate structures embedded within a broadly distributed nuclear signal and further support the spatial association detected by the colocalisation colormap analysis. Next, we wondered whether *HTT* nuclear RNA structures were enriched within other nuclear markers and organelles such as nucleolus (rRNA45S), Cajal bodies (coilin), paraspeckles (SFPQ), open state chromatin (H3K9ac), RNA nuclear export (p84) or different RNA-binding proteins (TDP-43, hnRNPF, hnRNPK). Analysis of the experimental data demonstrated that none of the tested markers colocalised with *HTT* nuclear RNA clusters (Supplementary Fig. 7). These data complement previous reports that several splicing factors, including PRPF8 and SNRNP40, can be trapped by mutant *HTT* RNA and further support the idea that these interactions might play a role in the alternative processing and production of *HTT1a*.

4. The authors conclude that RNA clusters were confined to neurons, implying a neuron-specific mechanism of accumulation. "The FL-HTT probe revealed large RNA clusters detained in the cells of neuronal origin but not in the glial cells which mainly contained single FL-HTT mRNAs (Supplementary Fig. 2b)." This conclusion is not supported by the data shown on Supplementary Fig 2b. It is not possible to distinguish large clusters from single mRNAs from the images shown. Images at higher

magnification and some kind of quantitation should be included to support this. The same issue in Figure 4C- the data need to be quantitated to draw the conclusion.

We have now included higher-magnification images in the revised Supplementary Figure 2b and Fig. 4d where *HTT* RNA nuclear clusters are readily observable in **NeuN-positive neurons** (yellow arrowheads**)**, while **GFAP-positive glial cells** (white arrowheads) contain single punctate *FL-HTT* mRNA molecules**.**

5. Images on Fig 6a are not representative for HTT intron 1 and 3 as similar few RNA clusters are visible in treated and control cells, whereas quantitative data shows a drastic decrease with 10h treatment.

We thank the reviewer for this comment. We have now included a different set of images that represent the data better.

6.The inset position is not clearly visible in most figures

We apologise for this inconvenience and have now modified the inset so that it is more evident.

7.The following statement in the abstract **-**"Huntington's disease research has focused on a toxic protein gain-of-function as the main driver of pathology"-does not consider the loss of normal function of HTT as potential mechanism, suggested by several studies. This should be noted.

We thank the reviewer for this remark. We have now modified this sentence in the abstract (lines 8-11), which now reads: Huntington's disease research has focused on either loss of normal huntingtin function or toxic gain-of-function of the mutant huntingtin protein as a key driver of pathology

8.The "Conclusion" section does not really summarize conclusions directly arising from the data shown, but rather present hypotheses of HTT RNA toxicity based on previous studies. These thoughts would be more appropriate within general Discussion or Introduction, unless the authors can directly connect their data with proposed conclusions.

We thank the reviewer for this comment. We have now modified the conclusion section so that it better summarises the data included in the current manuscript (lines 472-486).

The revised text is:

Conclusions

We show that *HTT* nuclear RNA clusters can be detected in YAC128 embryos and that they persist into late adulthood (up to 12 months), although their composition may

change over time. These structures are predominantly observed in neurons rather than glia and are dynamic, as their integrity is disrupted by the inhibition of ionic interactions, transcription, or splicing. The functional significance of these clusters remains unclear. Nuclear retention of mutant *HTT* mRNA could potentially limit the production of toxic protein isoforms; however, expanded CAG-repeat RNA itself has been implicated in polyQ pathogenesis through the formation of hairpin structures that sequester key RNA-binding proteins, such as MBNL1, leading to splicing dysregulation, as observed in myotonic dystrophy. Whether *HTT* RNA clusters interact with MBNL1 remains unresolved, as available antibodies did not yield a detectable signal. Notably, published data suggest that *HTT* nuclear clusters are resistant to ASO treatment, highlighting the need to define their function and determine whether they occur in other HD models, including human tissue, to inform the development of RNA-targeted therapeutics.

Reviewer #2 (Remarks to the Author):

This manuscript characterizes HTT RNA nuclear clusters in the brains of pre- and post-natal YAC128 mouse brains, and in primary mixed cultures. The authors show the clusters are present in all tested model systems, and at all tested developmental time points. The manuscript claims the clusters may contribute to HD pathophysiology by disrupting RNA splicing in neurons, and may inform targeting strategies used by future HD therapeutics. The manuscript demonstrates that HTT RNA nuclear clusters are detectable in prenatal HD mouse model brains, which is a novel finding that contributes to the field's understanding of HD pathogenesis. In particular, the developmental aspect of HD biology is relatively understudied, and this manuscript will make a valuable contribution to this area of HD research. Association of the clusters with splicing factor PRPF8 also adds to existing work from this group that explores aberrant splicing of HTT mRNA as a contributor to HD pathophysiology. The impact of CAG-repeat containing mRNA on nuclear dynamics may also be of interest to wider audiences focused on other CAG repeat disorders, as the authors mention in the text. In general, the manuscript is logically organized, the experiments are explained well, and descriptions of the statistics are thorough and the tests used are appropriate. The conclusions of the work are original (although a bit preliminary). This work elucidates a novel aspect of HD pathophysiology that contributes to the growing body of evidence suggesting RNA toxicity is important in HD. It is likely this work will support further investigation of RNA-associated disease mechanisms in the HD field.

1.The manuscript would benefit from general editing to improve readability and address minor grammar and punctuation errors.

We thank the reviewer for this helpful suggestion. We have carefully revised the manuscript to improve overall readability and have corrected minor grammatical and punctuation errors throughout.

2.The authors present a number of hypotheses in the discussion exploring mechanistic drivers of RNA cluster formation and stability; however, these are not followed up or explored with experiments in the manuscript, and doing so would increase the novelty and impact of the work.
For example, m6A modification could be measured and correlated with RNA cluster dynamics in pre- and post-natal mice.

We thank the reviewer for this observation. We have now performed RNA–IF experiments using an antibody against N6-methyladenosine (m^6A) (please see below). While a signal is detectable, it is predominantly cytoplasmic, consistent with the fact that the majority of methylated mRNAs accumulate in the cytoplasm. A weaker signal can be observed in the nucleus; however, nuclear m^6A staining is typically **low in abundance and highly punctate**, as methylation occurs in small subnuclear compartments. Consequently, nuclear detection by IF is inherently challenging. In addition, antibody-based detection of RNA modifications reports the **global pool of methylated RNAs**, rather than modification of a specific transcript. In neuronal nuclei this results in a diffuse background signal that makes it difficult to determine whether methylation originates specifically from *HTT* RNA clusters. Furthermore, the structural

accessibility of these clusters is likely limited. Because they are located near sites of active RNA processing, they may be associated with chromatin and RNA–protein complexes, which can lead to **epitope masking and reduced antibody accessibility**, further limiting the sensitivity of IF detection. For these reasons, we do not consider IF to be the most reliable approach to assess methylation of *HTT* RNA clusters. Methods that directly interrogate RNA modifications on specific transcripts, such as **m^6A profiling approaches (e.g., MeRIP-seq / m^6A-seq)**, would provide a more informative and transcript-specific assessment, however, are currently outside the scope of this study.

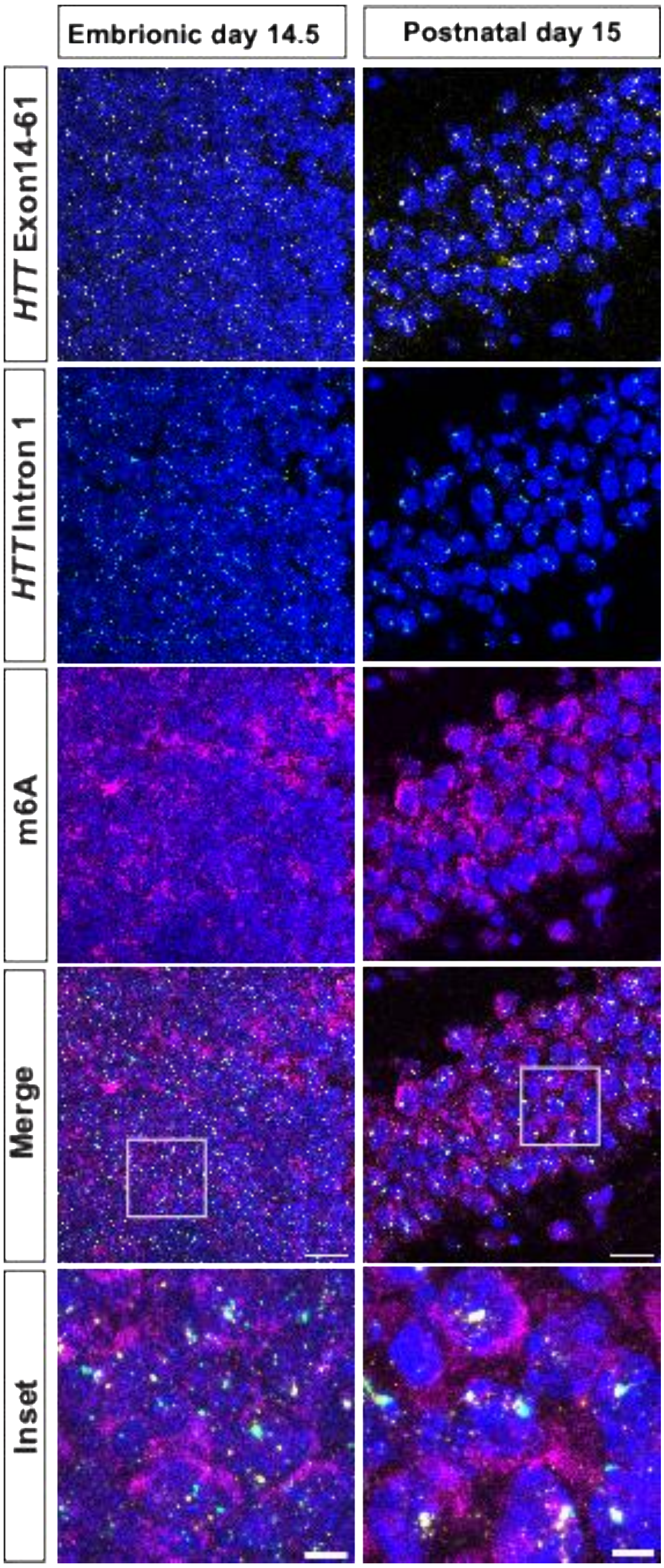

3. The impact of destabilizing the clusters upon an HD-associated cellular phenotype could also be assessed to explore the putative contribution of HTT RNA clusters to HD pathogenesis.

We thank the reviewer for this comment. While we agree that investigating the effect of clusters disassembly on HD-associated phenotypes would be valuable, these experiments are currently not feasible. We can provide at least two explanations:

1. HD cellular models (unless an exogenous huntingtin construct in expressed) do not show robust HD-associated phenotypes that can be easily interrogated. (e.g aggregation, mitochondria deficits). This is especially true for neuronal cultures derived from P0 pups as e.g protein aggregation and mitochondria impairment are associated with later stages of HD.
2. The agents used in the study have pleiotropic effects and it would be very difficult to unpick whether a certain phenotype or lack thereof stems from the treatment itself or from the disintegration of the clusters.

4. The introduction references the presence of nuclear HTT RNA clusters in the brains of YAC128 and BACHD mice, and states that this evidence indicates the RNA clusters only form when human huntingtin transcripts are present. Has a study been done that shows these clusters aren't present in a full-length murine KI HD model (e.g., the Hdh(CAG)150 model)? If so, it should be referenced here. If not, this study should be conducted, or the claims in this section should be tempered to accurately reflect existing data.

We thank the reviewer for this comment. Indeed, we have shown in our previous paper (Fienko et all, Brain, 2022) that RNA clusters only form in YAC128 brains that harbours full-length human huntingtin allele, as opposed to zQ175 knock-in HD mouse model. In zQ175 animals (where mouse exon1 has been replaced with the human exon 1 with -180 CAG under mouse *Htt* promoter) only single huntingtin transcripts were detected, and these mice showed no evidence of cluster formation (**Fienko et all, Brain, 2022**; **Supplementary Fig 5**). Additionally, unpublished data from our lab revealed no evidence of cluster formation in the *Hdh*Q111 and *Hdh*Q107 HD mouse models.

5.The volume ranges used to distinguish between single nuclear mRNA transcripts and RNA clusters overlap. Please modify the bin boundaries to correct this.

We thank the review for this comment; we have now modified the bin boundaries accordingly.

6. It would be helpful to fully label the y-axes in Fig. 1c-e, as the lower bounds of the second and third y-axis segments are not numbered.

We thank the review for this comment; we have now labelled the y-axis accordingly.

7. It also appears that the measurements reported in the text don't match the plotted measurements in this figure (e.g., early postnatal and adult mice appear to have FL-HTT clusters that measure <40 um^3 based on the plotted data, but the text reports these measurements to reach up to 45 um^3). Similarly, the largest clusters measured in E14.5 mice (in Fig. 1c) all appear to be <10 um^3. The data distribution is also different in the corresponding supplementary figure, but it is unclear what the difference is between the two figures (e.g., it is reported that the primary figure shows averages, but how were these data computed?).

We thank the reviewer for this insightful observation. We have now clarified the analysis procedure in the manuscript (lines 105-120).

Briefly, the volumes of both RNA clusters and single HTT mRNA molecules were quantified across all analysed brain sections. The measured volumes were then grouped into three size bins to facilitate comparison between developmental stages. For the analysis shown in Fig. 1 and 2, measurements were first averaged within each bin for a given brain section, and these values were then averaged across sections from the same animal to generate a single value per bin per mouse. The values plotted in **Fig. 1** therefore represent **animal-level averages**. In contrast, **Supplementary Fig. 3** shows the **distribution of all individual cluster measurements** obtained across all brain sections analysed in the RNAscope experiment. Because this plot includes the full spread of individual measurements, it contains occasional larger clusters (e.g., up to ~45 μm³), whereas the averaged values plotted in Fig. 1 fall within a lower range (approximately ~25 μm³ for the corresponding bin). The same principle applies to the measurements obtained at E14.5 and at other developmental stages.

The revised text is:

Figures 1 and 2 show animal-level averages for each of these three bins, whereas Supplementary Fig. 3 depicts the full distribution of all individual cluster and transcript measurements obtained across the analysed sections. *The FL-HTT* probe revealed large RNA clusters detained in the cells of neuronal origin but not in the glial cells which mainly contained single *FL-HTT* mRNAs (Supplementary Figure 2b). Huntingtin nuclear RNA clusters as well as single *FL-HTT* transcripts present in the nucleus and cytoplasm were detected in embryonic, postnatal and adult YAC128 mice (Fig. 1b,c). The biggest *FL-HTT* clusters detected in the early postnatal and adult mice reached up to 45 μm^3, whereas in mice at E14.5 these clusters were significantly smaller with the largest clusters approaching 17 μm^3 in volume (Supplementary Figure 3a). Interestingly, there was a marked increase in the average volume of the smaller *FL-HTT* clusters classified in the 2nd bin (2–20 μm^3), between early postnatal age P1 and later postnatal stage P15, and then a sharp decrease in 12-month-old mice, suggesting a dynamic regulation of cluster size across developmental and aging stages (Fig. 2a, b).

8. There are also >3 data points for each animal group, but N=3 is reported in the figure caption.

We apologize for the confusion. The reported n = 3 refers to the number of animals used in the study. From each animal, multiple brain sections were processed and stained using the RNAscope procedure. The individual data points initially shown in the graphs therefore corresponded to measurements obtained from individual sections rather than independent biological replicates. We recognize that presenting these values could give the impression of oversampling. To address this, we have now averaged the measurements from sections derived from each animal and updated the graphs accordingly, so that each data point represents one animal (n=3*).*

9. On page 4, the authors state size changes in RNA clusters over time suggest "a dynamic regulation of cluster size across developmental and aging stages". This statement suggests this is an active process/mechanism that regulates cluster size in YAC128 brain over time. Is this accurate or could cluster size fluctuations be a consequence of other processes?

We thank the reviewer for this insightful comment. Indeed, many processes could contribute to the fluctuations in the size of the huntingtin nuclear clusters. Currently, it is difficult to point towards one mechanism and given the complexity and various molecular changes that arise during development and aging, we cannot exclude a crosstalk of multiple processes. Possible explanations with regards to changes in the *HTT* cluster size include differences in the transcription rate or RNA turnover (including on the global scale); divergent RNA-binding protein repertoire or differential RNA modifications that govern RNA retention in embryo vs postnatal mice, changes in RNA structural dynamics or environmental changes.

10. In Fig. 4, the inset on the overview that indicates the sampled area in remaining panels is very difficult to see – could this be changed so the inset is easier to distinguish?

We apologise for this inconvenience and have now modified the inset so that it is more evident.

11. On page 8, a concluding sentence reads "these results suggest that HTT nuclear RNA cluster assembly is partly regulated by ionic interactions mediated by proteins that neutralize negatively charged RNA molecules". The first part of this statement is supported by the data, but there is not biological evidence to suggest proteins that interact with negatively-charged RNA mediate the formation of RNA nuclear clusters, as chemical treatments were used to manipulate cluster morphology. Please temper this claim or include an additional experiment that specifically addresses the biological mechanism(s) driving this observation.

We thank the reviewer for this comment and apologise for the lack of clarity. In biological systems, ionic interactions involving RNA are most commonly mediated by RNA-binding proteins, which can partially neutralize the negative charge of RNA and thereby promote intermolecular interactions. Because *HTT* nuclear clusters consist of

multiple RNA molecules and are likely associated with RNA-binding proteins, including PRPF8 as well as SNRNP40 and potentially other components of the RNA processing machinery, we suggested that protein-mediated ionic interactions could contribute to cluster assembly. However, we agree that our current experiments rely on chemical perturbations that affect ionic interactions globally and therefore do not directly demonstrate the specific involvement of RNA-binding proteins in this process. To avoid overstating this conclusion, we have now tempered the wording in the manuscript (lines 262-265).

The revised text is:

These results suggest that *HTT* nuclear RNA cluster assembly is partly regulated by ionic interactions, which in biological systems may be facilitated by RBPs that help neutralize the negative charge of RNA molecules.

12. The last sentence of the Results states that “elevated levels of HTT1a in the extranuclear space are indicative of its escape from nuclear retention and its accumulation in the cytoplasmic compartment”. However, the figures referenced in the previous sentences (Fig. 9c and Supp. Fig. 9f) show increases in HTT1a transcripts both inside and outside the nucleus – is this possible?

We thank the reviewer for this question and are happy to clarify our reasoning. Under control conditions, *HTT1a* transcripts are predominantly contained within clusters, with very few single mRNA molecules detected. Upon treatment with PladB, we observe an increase in single *HTT1a* transcripts in both the cytosolic and nuclear compartments. Inhibition of canonical splicing leads to aberrant RNA processing, resulting in an increased number of alternatively processed *HTT1a* transcripts within the nucleus. Concurrently, disassembly of *HTT1a* nuclear clusters may liberate single *HTT1a* transcripts, releasing them into the nucleoplasm. Both mechanisms likely contribute to the observed rise in nuclear *HTT1a* levels. These single nuclear *HTT1a* mRNA molecules subsequently become available for export into the cytoplasm, accounting for the increase in extranuclear *HTT1a* levels.
