# Supplementary material for: Nuclear RNA clusters are dynamic structural entities in Huntington’s disease
Source: Commun Biol. 2026 Jun 29;9:1370. doi: 10.1038/s42003-026-10419-1 (PMC13680494; doi:10.1038/s42003-026-10419-1)
Supplement: Supplementary file 3 [file 42003_2026_10419_MOESM3_ESM.pdf]

Description of Additional Supplementary Files

File name: Supplementary Data 1

Description: The source data behind the graphs in the paper

File name: Supplementary Data 2

Description: The source data behind the graphs in the paper
