# Supplementary material for: Nuclear RNA clusters are dynamic structural entities in Huntington’s disease
Source: Commun Biol. 2026 Jun 29;9:1370. doi: 10.1038/s42003-026-10419-1 (PMC13680494; doi:10.1038/s42003-026-10419-1)
Supplement: Supplementary file 4 [file 42003_2026_10419_MOESM6_ESM.pdf]

nature portfolio

Corresponding author(s): Sandra Fienko

Last updated by author(s): Apr 11, 2026

Reporting Summary

Nature Portfolio wishes to improve the reproducibility of the work that we publish. This form provides structure for consistency and transparency in reporting. For further information on Nature Portfolio policies, see our Editorial Policies and the Editorial Policy Checklist.

Statistics

For all statistical analyses, confirm that the following items are present in the figure legend, table legend, main text, or Methods section.

n/a	Confirmed	
☐	☒	The exact sample size (*n*) for each experimental group/condition, given as a discrete number and unit of measurement
☐	☒	A statement on whether measurements were taken from distinct samples or whether the same sample was measured repeatedly
☐	☒	The statistical test(s) used AND whether they are one- or two-sided<br>*Only common tests should be described solely by name; describe more complex techniques in the Methods section.*
☒	☐	A description of all covariates tested
☐	☒	A description of any assumptions or corrections, such as tests of normality and adjustment for multiple comparisons
☐	☒	A full description of the statistical parameters including central tendency (e.g. means) or other basic estimates (e.g. regression coefficient) AND variation (e.g. standard deviation) or associated estimates of uncertainty (e.g. confidence intervals)
☐	☒	For null hypothesis testing, the test statistic (e.g. *F*, *t*, *r*) with confidence intervals, effect sizes, degrees of freedom and *P* value noted<br>*Give P values as exact values whenever suitable.*
☒	☐	For Bayesian analysis, information on the choice of priors and Markov chain Monte Carlo settings
☒	☐	For hierarchical and complex designs, identification of the appropriate level for tests and full reporting of outcomes
☒	☐	Estimates of effect sizes (e.g. Cohen's *d*, Pearson's *r*), indicating how they were calculated

Our web collection on statistics for biologists contains articles on many of the points above.

Software and code

Policy information about availability of computer code

Data collection	Zeiss Blue 3.9
Data analysis	FIJI (Image J, V 2.14.0),plugins: FIJI 3D Objects Counter , Colocalisation Colormap; GraphPad Prism 10, Zeiss Blue 3.9

For manuscripts utilizing custom algorithms or software that are central to the research but not yet described in published literature, software must be made available to editors and reviewers. We strongly encourage code deposition in a community repository (e.g. GitHub). See the Nature Portfolio guidelines for submitting code & software for further information.

Data

Policy information about availability of data

All manuscripts must include a data availability statement. This statement should provide the following information, where applicable:

- Accession codes, unique identifiers, or web links for publicly available datasets
- A description of any restrictions on data availability
- For clinical datasets or third party data, please ensure that the statement adheres to our policy

All data supporting the findings of this study are available within the paper and its Supplementary Information.Raw data are available from the corresponding author on request.

Research involving human participants, their data, or biological material

Policy information about studies with human participants or human data. See also policy information about sex, gender (identity/presentation), and sexual orientation and race, ethnicity and racism.

Reporting on sex and gender	The study did not involve human participants
Reporting on race, ethnicity, or other socially relevant groupings	The study did not involve human participants
Population characteristics	The study did not involve human participants
Recruitment	The study did not involve human participants
Ethics oversight	The study did not involve human participants

Note that full information on the approval of the study protocol must also be provided in the manuscript.

Field-specific reporting

Please select the one below that is the best fit for your research. If you are not sure, read the appropriate sections before making your selection.

☒ Life sciences ☐ Behavioural & social sciences ☐ Ecological, evolutionary & environmental sciences

For a reference copy of the document with all sections, see nature.com/documents/nr-reporting-summary-flat.pdf

Life sciences study design

All studies must disclose on these points even when the disclosure is negative.

Sample size	No sample size calculation was performed for this study. Sample sizes were chosen based on previous calculations and analyses.
Data exclusions	No data were excluded
Replication	All experiments were performed with at least three or more biological replicates. n corresponds to the number of individual mice per experiment. For YAC128 neuronal cultured cell experiments at least two pups per litter were used. For each pup at least 5-8 images were acquired.
Randomization	Experimental groups were randomised to avoid litter and cage effects.
Blinding	Investigators were blinded to the treated/non-treated group when performing experiments involving YAC128 neuronal cultured cell treatments.

Reporting for specific materials, systems and methods

We require information from authors about some types of materials, experimental systems and methods used in many studies. Here, indicate whether each material, system or method listed is relevant to your study. If you are not sure if a list item applies to your research, read the appropriate section before selecting a response.

Materials & experimental systems

n/a	Involved in the study	
☐	☒	Antibodies
☒	☐	Eukaryotic cell lines
☒	☐	Palaeontology and archaeology
☐	☒	Animals and other organisms
☒	☐	Clinical data
☒	☐	Dual use research of concern
☒	☐	Plants

Methods

n/a	Involved in the study	
☒	☐	ChIP-seq
☒	☐	Flow cytometry
☒	☐	MRI-based neuroimaging

Antibodies

Antibodies used	For immunofluorescence the following anibodies were used:

Antibodies used	SNRNP40 (Atlas Antibodies, HPA038904), rabbit<br>NeuN (Abcam, ab177487), rabbit<br>GFAP (Invitrogen, PA1-10004), chicken<br>PRPF8 (Abcam,ab79237), rabbit<br>coilin (Abcam, ab11822), mouse<br>SFPQ (Abcam, ab38148), rabbit<br>H3K9ac (Upstate, cell signalling solutions06-942), rabbit<br>p84 (GeneTex, GTX118740), rabbit<br>TDP-43 (Abcam, ab104223), mouse<br>hnRNP K (Abcam, ab52600), rabbit<br>hnRNP F (Thermofisher Scientific, CF500804), mouse<br>Secondary antibodies included:<br>Alexa555, Invitrogen, A32773, anti-mouse<br>Alexa555, Invitrogen, A32794, anti-rabbit<br>Alexa488, Invitrogen, A11055, anti-goat
Validation	The antibodies listed have been extensively used and validated by the manufacturer.<br>NeuN:https://www.abcam.com/en-us/products/primary-antibodies/neun-antibody-epr12763-neuronal-marker-ab177487<br>GFAP:https://www.thermofisher.com/antibody/product/GFAP-Antibody-Polyclonal/PA1-10004<br>PRPF8:https://www.abcam.com/en-us/products/primary-antibodies/prpf8-prp8-antibody-ab79237<br>Coilin:https://www.abcam.com/en-us/products/primary-antibodies/coilin-antibody-pdelta-ab11822<br>SFPQ: https://www.abcam.com/en-us/products/primary-antibodies/sfpq-antibody-ab38148<br>Histone H3 (Lys9): https://www.sigmaaldrich.com/GB/en/product/mm/06942?<br>srsltid=AfmBOoqsmpv_TFoWnINNln7scNi4lbVzL_YN_t6XHlvjxVqIfi7PlUCv<br>p84: https://www.genetex.com/Product/Detail/Nuclear-Matrix-Protein-p84-antibody/GTX118740?<br>srsltid=AfmBOorChB2QSYpJy4UKRzDEpXr-COIvLuQTqKphZdsI1ruEqVrFsyXd<br>TDP43:https://www.abcam.com/en-us/products/primary-antibodies/tdp43-antibody-3h8-ab104223<br>hnRNP K: https://www.abcam.com/en-us/products/primary-antibodies/hnrnp-k-antibody-ep943y-ab52600<br>hnRNP F:https://www.thermofisher.com/antibody/product/HNRNPF-Antibody-clone-OTI5F5-Monoclonal/CF500804<br>SNRNP40:https://www.atlasantibodies.com/products/primary-antibodies/triple-a-polyclonals/anti-snrnp40-antibody-hpa026527-25ul/

Animals and other research organisms

Policy information about studies involving animals; ARRIVE guidelines recommended for reporting animal research, and Sex and Gender in Research

Laboratory animals	YAC128 mice were maintained on a C57BL6/J background. The following ages were used: embryonic day 14.5, postnatal day 15; 1,2 as well as 12-month old animals
Wild animals	No wild animals were used in the study.
Reporting on sex	An equal number of male and female animals were used in the study.
Field-collected samples	no field-collected samples were used.
Ethics oversight	All procedures were performed in accordance with the Animals (Scientific Procedures) Act, 1986, and approved by the University College London Ethical Review Process Committee.

Note that full information on the approval of the study protocol must also be provided in the manuscript.

Plants

Seed stocks	This study did not involve plants
Novel plant genotypes	This study did not involve plants
Authentication	This study did not involve plants
